# Cobalt-catalyzed nucleophilic addition of the allylic C(sp^3^)–H bond of simple alkenes to ketones

**DOI:** 10.3762/bjoc.14.176

**Published:** 2018-08-02

**Authors:** Tsuyoshi Mita, Masashi Uchiyama, Kenichi Michigami, Yoshihiro Sato

**Affiliations:** 1 Faculty of Pharmaceutical Sciences, Hokkaido University, Sapporo 060-0812, Japan

**Keywords:** alkenes, C–H activation, C(sp^3^)–H bonds, cobalt, ketones

## Abstract

We herein describe a cobalt/Xantphos-catalyzed regioselective addition of simple alkenes to acetophenone derivatives, affording branched homoallylic alcohols in high yields with perfect branch selectivities. The intermediate of the reaction would be a nucleophilic allylcobalt(I) species generated via cleavage of the low reactive allylic C(sp^3^)–H bond of simple terminal alkenes.

## Introduction

**Figure 1:**
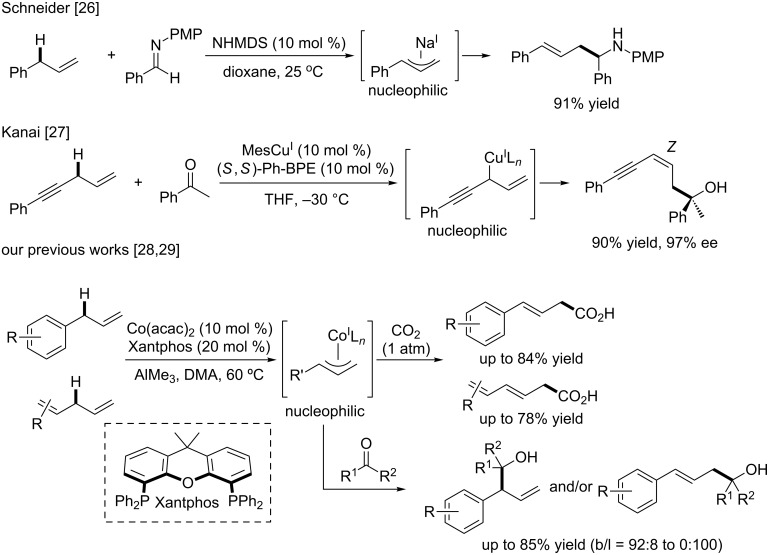
Precedent examples of catalytic allylic C(sp^3^)–H additions to carbonyl electrophiles.

The cleavage of C–H bonds of unreactive hydrocarbon followed by functionalization should be an ideal method for constructing complex molecules without introduction of reactive functionality in advance [1–9]. Since terminal alkenes including α-olefins (C_x_H_2x_) are abundantly present in nature or are readily accessible, they should be appropriate starting materials for C–C bond forming reactions to create organic frameworks of value-added compounds such as natural products, drugs, and fine chemicals. There have been tremendous synthetic methods involving catalytic C–C bond construction with the double bond of terminal alkenes (e.g., Heck reaction, hydrometalation followed by functionalization, carbometalation, and olefin metathesis) [10–13]. However, direct C–C bond formation of the allylic C(sp^3^)–H bond adjacent to double bonds has remained underdeveloped even though C–O bond formation of allylic C(sp^3^)–H bonds was firmly established by using SeO_2_ [14] or CrO_3_/3,5-dimethylpyrazole [15] (ene-type allylic oxidation). Although the most prominent work on catalytic allylic functionalization studied thus far is considered to be a palladium-catalyzed C–C bond formation using a stoichiometric amount of an oxidant [16–22], the π-allylpalladium intermediate [23–25] is an electrophilic species that exclusively reacts with nucleophiles. Therefore, it would be a formidable challenge for the generation of a nucleophilic π-allylmetal complex that reacts with electrophiles, triggered by allylic C(sp^3^)–H activation. To this end, Schneider [26], Kanai [27], and we [28–29] reported in 2017 catalytic allylic C(sp^3^)–H activation of alkenes to react carbonyl electrophiles such as imines, ketones, and CO_2_ via nucleophilic allylmetal species (Figure 1). However, the substrates employed have been restricted to allylarenes and 1,4-enyne, and 1,4-diene derivatives and α-olefins were totally unexplored. Therefore, the next challenge would be to use less reactive α-olefins (p*K*_a_ value of 1-propene = 43). In this paper, we describe an allylic C(sp^3^)–H addition of α-olefins, mainly 1-undecene and their analogues, to ketone electrophiles.

## Results and Discussion

We initially conducted screening of conditions using 1 equiv of 1-undecene (**1a**) and 3 equiv of acetophenone (**2a**) as starting materials (Table 1). When the reaction was conducted at 60 °C in DMA according to our previously established catalytic conditions (Co(acac)_2_ (10 mol %), Xantphos (20 mol %), and AlMe_3_ (1.0 equiv)) [29], branched homoallylic alcohol **3aa** was obtained in only 23% yield with 1.6:1 diastereoselectivity (Table 1, entry 1). In constant to the C(sp^3^)–H addition of allylarene to acetophenone that exhibited high linear selectivity [29], perfect branch selectivity was observed using **1a** as a substrate. When the reaction temperature was raised, the yield of **3aa** was improved to 45% yield at 90 °C (Table 1, entries 2 and 3). An increase in the amount of AlMe_3_ to 1.5 equiv further improved the yield of **3aa** to 54% yield (Table 1, entry 4). The moderate yield was attributed to the generation of the olefin isomerization product derived from **1a** (vide infra). We then changed the equivalents of reagents **1a** and **2a**. Although the yield of **3aa** was decreased when the reaction was conducted using a 1:1 ratio of **1a** and **2a** (Table 1, entry 5), the use of an excess amount of **1a** (3 equiv) greatly improved the yield to 70% (Table 1, entry 6: optimized conditions). When 1-octadecene (**1b**) was subjected to the optimized reaction conditions without adding **2a**, internal olefins were exclusively obtained (mixture of positional and geometric isomers) in 97% yield. It was shown by ^1^H NMR analysis that the mixture contained about 60% of 2-octadecene (*E*/*Z* mixture).

**Table 1:** Screening of reaction conditions.

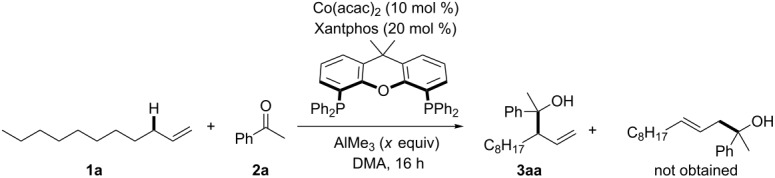				

Entry	**1a**:**2a**	Temp (°C)	AlMe_3_ (*x* equiv)	**3aa** (dr) (%)^a^

1	1:3	60	1.0	23 (1.6:1)
2	1:3	80	1.0	44 (1.3:1)
3	1:3	90	1.0	45 (1.3:1)
4^b^	1:3	90	1.5	54 (1.3:1)
5	1:1	90	1.5	46 (1.2:1)
6	3:1	90	1.5	70^c^ (1.3:1)

^a^Yields were determined by ^1^H NMR analysis using 1,1,2,2-tetrachloroethane as an internal standard. The diastereoselectivity (dr) was determined by ^1^H NMR analysis. ^b^The olefin isomerization product was obtained in 32% yield. ^c^Isolated yield.

**Figure 2:**
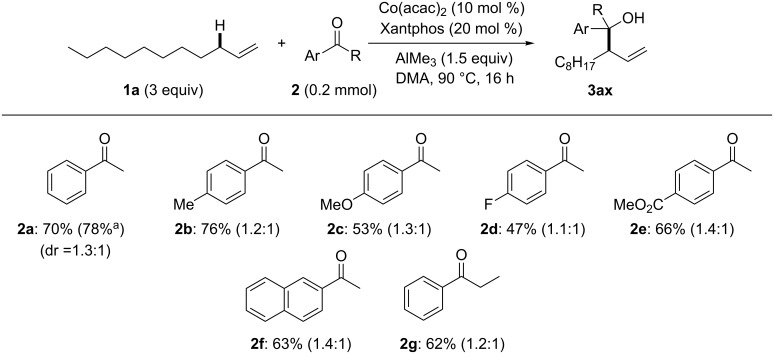
Substrate scope for acetophenone derivatives. ^a^Preparative scale synthesis using 1 mmol of **2a**.

Having established the reaction conditions, we then screened the scope and limitation of substituted acetophenone derivatives using an excess amount of 1-undecene (**1a**, 3 equiv, Figure 2). Electron-neutral and electron-donating substituents such as H (**2a**), Me (**2b**), and OMe (**2c**) at the *para*-position efficiently promoted the allylic C(sp^3^)–H addition, in which the reaction of **2a** could be scaled-up (1 mmol) to afford **3aa** in a slightly higher yield (78%). Electron-withdrawing substituents such as F (**2d**) and CO_2_Me (**2e**) also promoted the reaction with similar levels without damaging the ester functionality. Furthermore, 2-naphtophenone (**2f**) and propiophenone (**2g**) were tolerated well, affording branched products selectively in over 60% yield. However, *p*-CF_3_-acetophenone (18%), acetone (14%), cyclohexanone (29%), and benzophenone (25%) were not suitable substrates for C(sp^3^)–H addition of **1a** (figures not shown).

**Figure 3:**
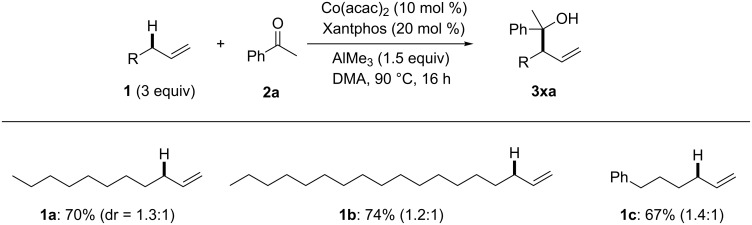
Substrate scope for α-olefins.

We next examined several α-olefins **1** (3 equiv) for allylic C(sp^3^)–H addition to acetophenone (**2a**). Not only 1-undecene (**1a**) but also 1-octadecene (**1b**) and 6-phenyl-1-hexene (**1c**) were tolerable to afford the corresponding products in around 70% yield with perfect branch selectivity (Figure 3). Although the allylic C(sp^3^)–H bond of α-olefins is weakly acidic (p*K*_a_ value of 1-propene = 43), it is noteworthy that thermal cleavage of allylic C(sp^3^)–H bonds is possible without using highly basic organolithium or organomagnesium reagents (Grignard reagents) that react with ketones rather than deprotonating the allylic C(sp^3^)–H bonds.

**Figure 4:**
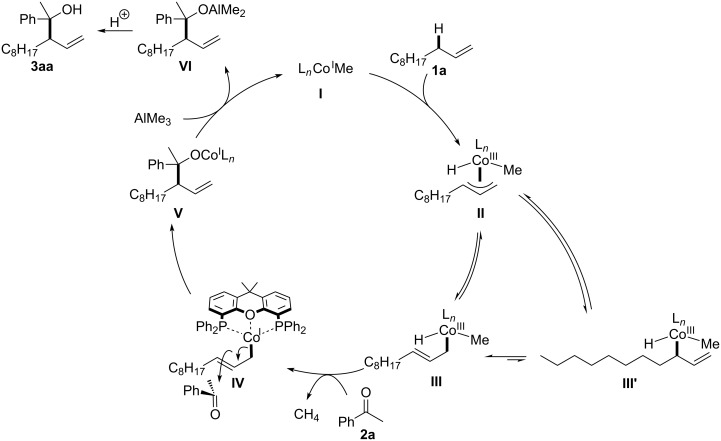
A possible catalytic cycle.

Based on the observed perfect branch selectivity, we propose the catalytic cycle of the C(sp^3^)–H addition of 1-undecene (**1a**) to acetophenone (**2a**, Figure 4). First, methylcobalt(I) **I** should be generated from Co(acac)_2_, Xantphos, and AlMe_3_ [28–29]. Oxidative addition of the allylic C(sp^3^)–H bond to **I** would proceed to afford η^3^-allylcobalt(III) intermediate **II**, which is tautomerized to η^1^-allylcobalt(III) **III** by the assistance of the oxygen atom in the Xantphos ligand [30]. When using α-olefin as a substrate, the cobalt atom should be located at the terminal position due to the avoidance of steric repulsion between the bulky Xantphos ligand and an alkyl substitution (similar to the case of nucleophilic η^1^-allylpalladium species [31–39]), whereas the cobalt atom preferred to reside at the internal position when allylarenes and 1,4-dienes were employed in our previous studies [28–29]. Subsequently, reductive elimination of methane from **III** would lead to a low-valent allylcobalt(I) species, and then C–C bond formation of **IV** with **2a** would proceed at the γ-position to produce cobalt alkoxide(I) **V** [28–29,31–39]. Transmetalation between **V** and AlMe_3_ would furnish branched aluminium alkoxide **VI** along with the regeneration of **I**. Alkoxide **VI** is converted to homoallylic alcohol **3aa** by usual work-up.

## Conclusion

In conclusion, we have successfully developed a cobalt-catalyzed nucleophilic addition of the C(sp^3^)–H bond of simple alkenes to ketones. This novel transformation could realize perfect branch selectivity for all substrates. Much effort toward the development of an asymmetric variant is ongoing. We are also conducting computational analysis to explain the observed perfect regioselectivity. These results will be reported in due course.

## Experimental

Representative procedure: To an oven-dried test tube was placed Co(acac)_2_ (5.2 mg, 20 μmol, 10 mol %) and Xantphos (23.1 mg, 40 μmol, 20 mol %) in DMA (2 mL). The resulting mixture was stirred at room temperature until the materials had been completely dissolved. After the solution had been cooled to 0 ºC, it was stirred for 1 minute, and then AlMe_3_ (2 M in toluene, 0.15 mL, 0.3 mmol, 1.5 equiv) was added. The dark green solution was stirred for another 1 minute, and then alkene **1** (0.6 mmol, 3.0 equiv) was added followed by the addition of ketone **2** (0.2 mmol, 1.0 equiv). The resulting mixture was stirred at 90 ºC for 16 h. After cooling the mixture to 0 ºC, the reaction was quenched by 1 M HCl aq and extracted with ethyl acetate (3 times). The combined organic layer was washed with brine and dried over Na_2_SO_4_. After the solids had been filtered off, the solvent was removed under reduced pressure and the residue was dried under vacuum to afford the crude mixture. The approximate yield of **3** was determined at this stage using 1,1,2,2-tetrachloroethane (δ = 6.1 ppm in CDCl_3_, 2H) as an internal standard. If the ketone remained, NaBH_4_ was added to convert it into the corresponding alcohol, which could be easily separated from **3** by silica-gel column chromatography. It was then purified by silica-gel column chromatography to afford the products **3**.

## Supporting Information

File 1 Experimental details and characterization data.
