# Supplementary material for: Cobalt-catalyzed nucleophilic addition of the allylic C(sp3)–H bond of simple alkenes to ketones
Source: Beilstein J Org Chem. 2018 Aug 2;14:2012–7. doi: 10.3762/bjoc.14.176 (PMC6122116; doi:10.3762/bjoc.14.176)
Supplement: File 1 — Experimental details and characterization data. [file Beilstein_J_Org_Chem-14-2012-s001.pdf]

Supporting Information

for

Cobalt-catalyzed nucleophilic addition of the allylic C(sp^3)–H bond of simple alkenes to ketones

Tsuyoshi Mita*, Masashi Uchiyama, Kenichi Michigami and Yoshihiro Sato*

Address: Faculty of Pharmaceutical Sciences, Hokkaido University, Sapporo 060-0812, Japan

Email: Tsuyoshi Mita: tmita@pharm.hokudai.ac.jp; Yoshihiro Sato: biyo@pharm.hokudai.ac.jp

*Corresponding author

Experimental details and characterization data

Table of Contents

(A) General S3

(B) Materials and methods S3

(B-1) Availability of ketones S3

(B-2) Preparation of alkenes S3

(C) General procedure for allylic C(sp^3)–H addition to ketones S4

(C-1) Examination of ketone electrophiles S4

(C-2) Examination of alkenes S7

(D) Copies of ^{1}H NMR and ^{13}C NMR spectra S9

(A) General

All manipulations were carried out under an atmosphere of argon or nitrogen unless otherwise noted. Infrared (IR) spectra were recorded on a JASCO FT/IR 460 Plus Fourier transform infrared spectrophotometer. NMR spectra were recorded on a JEOL ECA-500 spectrometer, operating at 500 MHz (^{1}H) or 125 MHz (^{13}C). Chemical shifts in $CDCl_3$ were reported in the scale relative to $CHCl_3$ (7.26 ppm) for ^{1}H NMR, and to $CDCl_3$ (77.0 ppm) for ^{13}C NMR as internal references, respectively. NMR data are reported as follows: chemical shifts, multiplicity (s: singlet, d: doublet, t: triplet, q: quartet, m: multiplet, br: broad signal), coupling constant (Hz), and integration. EI-HRMS and ESI-HRMS spectra were measured on a JEOL JMS-T100GCv and Thermo Scientific Exactive, respectively. Gel permeation chromatography was performed on HPLC LC-9201 (Japan Analytical Industry Co., Ltd). Column chromatography was performed with Wakogel® FC-40 (20–40 μm, spherical, neutral). DMA was distilled from CaH_2. $AlMe_3$ (2.0 M in toluene) was purchased from Sigma-Aldrich Co. LLC. $Co(acac)_2$ and Xantphos were purchased from Tokyo Kasei, Co., Ltd.

(B) Materials and methods

(B-1) Availability of ketones

Acetophenone (**2a**) was purchased Nacalai Tesque. Inc. 4'-Methylacetophenone (**2b**) was purchased from Wako Pure Chemical Industries, Ltd. 4'-Methoxyacetophenone (**2c**) and 2-naphtophenone (**2f**) were purchased from Tokyo Kasei, Co., Ltd. 4'-Fluoroacetophenone (**2d**) was purchased from Sigma-Aldrich Co. LLC. Methyl 4-acetylbenzoate (**2e**) was purchased from Acros Organics Inc. Propiophenone (**2g**) was purchased from Kanto Chemical Co., Inc. All ketones except **2e** and **2f** were purified by distillation before use. **2e** and **2f** were used as received.

(B-2) Preparation of alkenes

1-Undecene (**1a**) and 1-octadecene (**1b**) were purchased from Tokyo Kasei, Co., Ltd and used after distillation. 6-Phenyl-1-hexene (**1c**) was prepared according to the reported procedure.[1]

---

[1] Krishna, P. R.; Srinivas, R. *Tetrahedron Lett.* **2007**, *48*, 2013.

(C) General procedure for allylic C(sp^3)–H addition to ketones

(C-1) Examination of ketone electrophiles

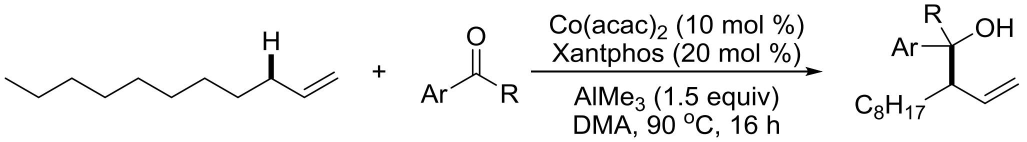

1a (3 equiv) + **2** (0.2 mmol) → **3ax**

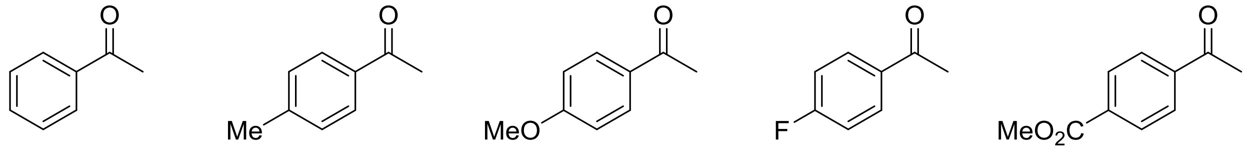

2a: 70% (78%[a]) (dr =1.3:1) **2b**: 76% (1.2:1) **2c**: 53% (1.3:1) **2d**: 47% (1.1:1) **2e**: 66% (1.4:1)

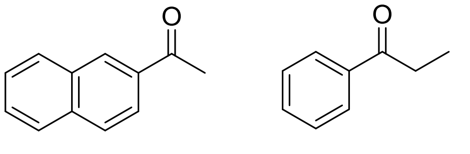

2f: 63% (1.4:1) **2g**: 62% (1.2:1)

[a]With 1 mmol of **2a**.

To an oven-dried test tube was placed Co(acac)$_2$ (5.2 mg, 20 μmol, 10 mol %) and Xantphos (23.1 mg, 40 μmol, 20 mol %) in DMA (2 mL). The resulting mixture was stirred at room temperature until the materials had been completely dissolved. After the solution had been cooled to 0 ºC, it was stirred for 1 minute, and then AlMe$_3$ (2 M in toluene, 0.15 mL, 0.3 mmol, 1.5 equiv) was added. The dark green solution was stirred for another 1 minute, and then 1-undecene (**1a**, 0.6 mmol, 120 μL, 3.0 equiv) was added followed by the addition of ketone **2** (0.2 mmol, 1.0 equiv). The resulting mixture was stirred at 90 ºC for 16 h. After cooling the mixture to 0 ºC, the reaction was quenched by 1 M HCl aq and extracted with ethyl acetate (3 times). The combined organic layer was washed with brine and dried over Na$_2$SO$_4$. After the solids had been filtered off, the solvent was removed under reduced pressure and the residue was dried under vacuum to afford the crude mixture. The approximate yield of **3ax** was determined at this stage using 1,1,2,2-tetrachloroethane (δ = 6.1 ppm in CDCl$_3$, 2H) as an internal standard. If ketone **2** remained, NaBH$_4$ was added to convert it into the corresponding alcohol, which could be easily separated from **3ax** by silica-gel column chromatography. It was then purified by silica-gel column chromatography to afford the product **3ax**.

2-Phenyl-3-vinylundecan-2-ol (3aa): **1a** (120 μL, 0.6 mmol, 3.0 equiv) and **2a** (23 μL, 0.2 mmol, 1.0 equiv) were employed as starting materials. Purification of the crude product by silica-gel column chromatography (eluent: hexane/ethyl acetate, 20:1) afforded **3aa** as colorless oil (38.5 mg, 140 μmol, 70%, dr = 1.3:1). Preparative scale synthesis: **1a** (600 μL, 3 mmol, 3.0 equiv), **2a** (117 μL, 1 mmol, 1.0 equiv), Co(acac)$_2$ (25.7 mg, 0.1 mmol, 10 mol %), Xantphos (115.7 mg, 0.2 mmol, 20 mol %), and AlMe$_3$ (2 M in toluene, 0.75 mL, 1.5 mmol, 1.5 equiv) were used in DMA (10 mL). Purification of the crude product by silica-gel column chromatography (eluent: hexane/ethyl acetate, 20:1) afforded 3aa as colorless oil (215.1 mg, 0.784 mmol, 78%, dr = 1.3:1).

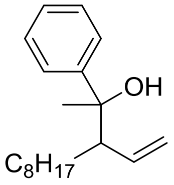

3aa

IR (neat): 3467, 2925, 2855, 1637, 1446, 1375, 1065, 913, 759, 701 cm^{-1}; ^{1}H NMR (400 MHz, CDCl$_3$) δ: 7.44-7.21 (m, 5H), 5.63-5.52 (m, 1H), 5.21-5.01 (m, 2H), 2.30-2.23 (m, 1H), 1.53/1.51 (s, 3H), 1.26-1.06

(m, 14H), 0.85 (t, *J* = 7.1 Hz, 3H) ppm; ^{13}C NMR (100 MHz, $CDCl_3$) δ: 147.2, 146.5, 138.9, 138.6, 127.84, 127.75, 126.6, 126.3, 125.9, 125.2, 118.8, 118.1, 76.0, 75.5, 56.6, 55.6, 31.8, 29.42, 29.41, 29.37, 29.2, 28.9, 28.6, 28.4, 27.8, 27.7, 25.4, 22.6, 14.1 ppm; HRMS (EI) *m/z* calcd. for $C_{19}H_{28}$ $[M-H_2O]^+$: 256.2191, found: 256.2191.

2-(*p*-Tolyl)-3-vinylundecan-2-ol (3ab): 1a (120 μL, 0.6 mmol, 3.0 equiv) and **2b** (27 μL, 0.2 mmol, 1.0 equiv) were employed as starting materials. Purification of the crude product by silica-gel column chromatography (eluent: hexane/ethyl acetate, 100:1 to 20:1) afforded **3ab** as colorless oil (44 mg, 153 μmol, 76%, dr = 1.2:1). IR (neat): 3466, 2925, 2855, 1637, 1512, 1457, 1374, 1078, 912, 817 cm^{-1}; ^{1}H NMR (400 MHz, $CDCl_3$) δ: 7.31/7.26 (d, *J* = 8.3 Hz, 2H), 7.14 (d, *J* = 8.3 Hz, 2H), 5.63-5.52 (m, 1H), 5.21-5.03 (m, 2H), 2.34 (s, 3H), 2.27-2.22 (m, 1H), 1.51/1.49 (s, 3H), 1.25-1.06 (m, 14H), 0.85 (t, *J* = 7.0 Hz, 3H) ppm; ^{13}C NMR (100 MHz, $CDCl_3$) δ: 144.2, 143.6, 139.1, 138.7, 136.1, 135.8, 128.54, 128.46, 125.8, 125.1, 118.7, 117.9, 75.8, 75.3, 56.6, 55.6, 31.8, 29.45, 29.43, 29.42, 29.39, 29.2, 28.9, 28.7, 28.5, 27.8, 27.7, 25.4, 22.6, 20.94, 20.91, 14.1 ppm; HRMS (EI) *m/z* calcd. for $C_{20}H_{30}$ $[M-H_2O]^+$: 270.2348, found: 270.2349.

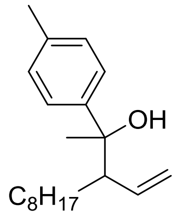

3ab

2-(4-Methoxyphenyl)-3-vinylundecan-2-ol (3ac): 1a (120 μL, 0.6 mmol, 3.0 equiv) and **2c** (27.5 mg, 0.2 mmol, 1.0 equiv) were employed as starting materials. Purification of the crude product by silica-gel column chromatography (eluent: hexane/ethyl acetate, 50:1) afforded **3ac** as colorless oil (29 mg, 96 μmol, 53%, dr = 1.3:1). IR (neat): 3466, 2925, 2854, 1611, 1510, 1248, 1179, 1036, 912, 832 cm^{-1}; ^{1}H NMR (400 MHz, $CDCl_3$) δ: 7.35 (d, *J* = 8.5 Hz, 1H), 7.29 (d, *J* = 8.5 Hz, 1H), 6.86 (d, *J* = 8.5 Hz, 2H), 5.61-5.52 (m, 1H), 5.22-5.03 (m, 2H), 3.81 (s, 3H), 2.26-2.20 (m, 1H), 1.51/1.49 (s, 3H), 1.26-1.00 (m, 14H), 0.85 (t, *J* = 7.0 Hz, 3H) ppm; ^{13}C NMR (100 MHz, $CDCl_3$) δ: 158.2, 158.0, 139.3, 139.1, 138.7, 127.1, 126.4, 118.8, 118.0, 113.1, 113.0, 75.7, 75.2, 56.8, 55.8, 55.2, 31.84, 31.82, 29.5, 29.4, 29.2, 28.82, 28.79, 28.5, 27.8, 27.7, 25.3, 22.6, 14.1 ppm; HRMS (EI) *m/z* calcd. for $C_{20}H_{30}O$ $[M-H_2O]^+$: 286.2297, found: 286.2292.

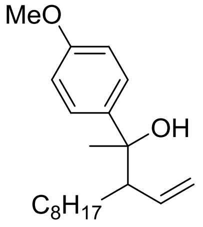

3ac

2-(4-Fluorophenyl)-3-vinylundecan-2-ol (3ad): 1a (120 μL, 0.6 mmol, 3.0 equiv) and **2d** (25.7 mg, 0.2 mmol, 1.0 equiv) were employed as starting materials. Purification of the crude product by silica-gel column chromatography (eluent: hexane/ethyl acetate, 20:1 to 10:1) afforded **3ad** as colorless oil (26 mg, 88 μmol, 47%, dr = 1.1:1). IR (neat): 3466, 2926, 2855, 1603, 1509, 1226, 1161, 1077, 915, 837 cm^{-1}; ^{1}H NMR (400 MHz, $CDCl_3$) δ: 7.41-7.31 (m, 2H), 7.04-6.98 (m, 2H), 5.60-5.50 (m, 1H), 5.23-5.03 (m, 2H), 2.25-2.18 (m, 1H), 1.56 (s, 1H), 1.52/1.50 (s, 3H), 1.29-1.00 (m, 14H), 0.86 (t, *J* = 7.0 Hz, 3H) ppm; ^{13}C NMR (100 MHz, $CDCl_3$) δ: 161.7 (d, J_{CF} = 248 Hz), 161.5 (d, J_{CF} = 249 Hz), 142.9 (d, J_{CF} = 2.8 Hz), 142.3 (d, J_{CF} = 2.9Hz), 138.8, 138.3, 127.6 (d, J_{CF} = 7.6 Hz), 126.9 (d, J_{CF}= 7.6 Hz), 119.2, 118.3, 114.52 (d, J_{CF} = 21 Hz), 114.46 (d, J_{CF} = 22 Hz), 75.7, 75.2, 56.8, 55.7, 31.8, 29.42, 29.40, 29.38, 29.2, 28.9, 28.7, 28.5, 27.7, 27.6, 25.3, 22.6, 14.0 ppm; HRMS (EI) *m/z* calcd. for $C_{19}H_{27}F$ $[M-H_2O]^+$: 274.2097, found: 274.2094.

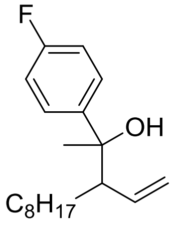

3ad

Methyl 4-(2-Hydroxy-3-vinylundecan-2-yl)benzoate (3ae): 1a (120 μL, 0.6 mmol, 3.0 equiv) and **2e** (36.9 mg, 0.2 mmol, 1.0 equiv) were employed as starting materials. Purification of the crude product by silica-gel column chromatography (eluent: hexane/ethyl acetate, 20:1 to 10:1) afforded **3ae** as colorless oil (46 mg, 139 μmol, 66%, dr = 1.4:1). IR (neat): 3500, 2926, 2855, 1726, 1609, 1437, 1281, 1191, 1115, 713 cm^{-1}; ^{1}H NMR (400 MHz, $CDCl_3$) δ: 8.00 (dd, *J* = 8.6, 1.4 Hz, 2H), 7.51-7.45 (m, 2H), 5.63-5.49 (m, 1H), 5.23-5.06 (m, 2H), 3.91 (s, 3H), 2.30-2.22 (m, 1H), 1.51/1.52 (s, 3H), 1.36-1.03 (m, 14H), 0.85 (t, *J* = 6.8 Hz, 3H) ppm; ^{13}C NMR (100 MHz, $CDCl_3$) δ: 167.0, 152.6, 151.9, 138.4, 138.0, 129.2, 129.1, 128.5, 128.2, 126.0, 125.3, 119.2, 118.5, 76.1, 75.6, 56.5, 55.4, 52.0, 31.8, 29.38, 29.36, 29.31, 29.2, 28.9, 28.5, 28.4, 27.7, 27.6, 25.5, 22.6, 14.1 ppm; HRMS (EI) *m/z* calcd. for $C_{21}H_{33}O_3$ $[M]^+$: 333.2430, found: 333.2423.

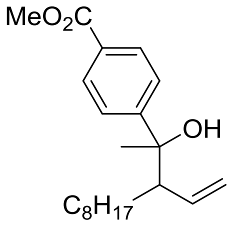

3ae

2-(Naphthalen-2-yl)-3-vinylundecan-2-ol (3af): 1a (120 μL, 0.6 mmol, 3.0 equiv) and **2f** (33.4 mg, 0.2 mmol, 1.0 equiv) were employed as starting materials. Purification of the crude product by silica-gel column chromatography (eluent: hexane/ethyl acetate, 50:1) afforded **3af** as colorless oil (40 mg, 123 μmol, 63%, dr = 1.4:1). IR (neat): 3466, 2925, 2854, 1636, 1464, 1375, 1127, 913, 817, 749 cm^{-1}; ^{1}H NMR (400 MHz, $CDCl_3$) δ: 7.87-7.78 (m, 4H), 7.59-7.41 (m, 3H), 5.69-5.55 (m, 1H), 5.22-5.07 (m, 2H), 2.40-2.33 (m, 1H), 1.62/1.57 (s, 3H), 1.26-1.02 (m, 14H), 0.81 (t, *J* = 7.0 Hz, 3H) ppm; ^{13}C NMR (100 MHz, $CDCl_3$) δ: 144.7, 144.1, 138.9, 138.5, 133.0, 132.9, 132.3, 132.1, 128.2, 128.1, 127.5, 127.44, 127.41, 125.9, 125.8, 125.67, 125.58, 124.56, 124.54, 124.48, 123.8, 123.7, 119.0, 118.2, 76.3, 75.7, 56.4, 55.3, 31.79, 31.78, 29.44, 29.39, 29.3, 29.2, 29.1, 28.7, 28.5, 27.8, 27.7, 25.4, 22.6, 14.1 ppm; HRMS (EI) *m/z* calcd. for $C_{23}H_{30}$ $[M-H_2O]^+$: 306.2348, found: 306.2345.

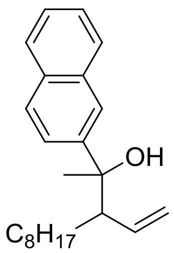

3af

3-Phenyl-4-vinyldodecan-3-ol (3ag): 1a (120 μL, 0.6 mmol, 3.0 equiv) and **2g** (27 μL, 0.2 mmol, 1.0 equiv) were employed as starting materials. Purification of the crude product by silica-gel column chromatography (eluent: hexane/ethyl acetate, 50:1) afforded **3ag** as colorless oil (36 mg, 125 μmol, 62%, dr = 1.2:1). IR (neat): 3495, 2925, 2855, 1637, 1463, 1376, 1002, 968, 913, 701 cm^{-1}; ^{1}H NMR (400 MHz, $CDCl_3$) δ: 7.38-7.30 (m, 4H), 7.25-7.20 (m, 1H), 5.64-5.43 (m, 1H), 5.21-5.02 (m, 2H), 2.33-2.23 (m, 1H), 2.10-2.02/1.87-1.74 (m, 2H), 1.25-0.95 (m, 14H), 0.88-0.83 (m, 3H), 0.77/0.66 (t, *J* = 7.5 Hz, 3H) ppm; ^{13}C NMR (100 MHz, $CDCl_3$) δ: 144.7, 144.1, 138.9, 138.7, 127.8, 127.6, 126.5, 126.3, 126.2, 125.8, 117.98, 117.96, 78.6, 78.1, 55.8, 54.8, 33.5, 31.8, 31.0, 29.6, 29.5, 29.4, 29.33, 29.26, 29.2, 28.3, 27.88, 27.78, 27.6, 22.64, 22.62, 14.1, 7.63, 7.58 ppm; HRMS (EI) *m/z* calcd. for $C_{20}H_{30}$ $[M-H_2O]^+$: 270.2348, found: 270.2347.

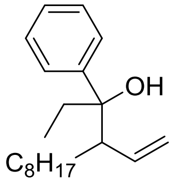

3ag

(C-2) Examination of alkenes

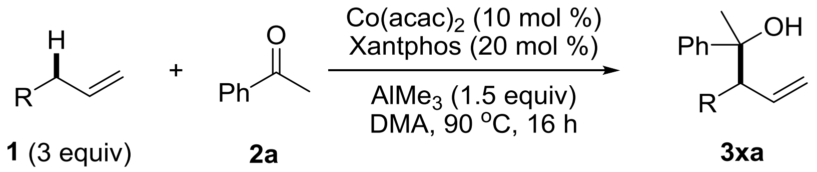

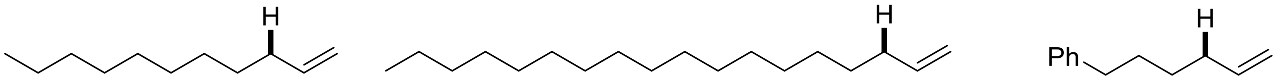

1a: 70% (dr = 1.3:1) **1b**: 74% (1.2:1) **1c**: 67% (1.4:1)

To an oven-dried test tube was placed Co(acac)$_2$ (5.2 mg, 20 μmol, 10 mol %) and Xantphos (23.1 mg, 40 μmol, 20 mol %) in DMA (2 mL). The resulting mixture was stirred at room temperature until the materials had been completely dissolved. After the solution had been cooled to 0 ºC, it was stirred for 1 minute, and then AlMe$_3$ (2 M in toluene, 0.15 mL, 0.3 mmol, 1.5 equiv) was added. The dark green solution was stirred for another 1 minute, and then alkene **1** (0.6 mmol, 3.0 equiv) was added followed by the addition of acetophenone (**2a**, 0.2 mmol, 23 μL, 1.0 equiv). The resulting mixture was stirred at 90 ºC for 16 h. After cooling the mixture to 0 ºC, the reaction was quenched by 1 M HCl aq and extracted with ethyl acetate (3 times). The combined organic layer was washed with brine and dried over Na$_2$SO$_4$. After the solids had been filtered off, the solvent was removed under reduced pressure and the residue was dried under vacuum to afford the crude mixture. The approximate yield of **3xa** was determined at this stage using 1,1,2,2-tetrachloroethane (δ = 6.1 ppm in CDCl$_3$, 2H) as an internal standard. If acetophenone (**2a**) remained, NaBH$_4$ was added to convert it into the corresponding 1-phenylethyl alcohol, which could be easily separated from **3xa** by silica-gel column chromatography. It was then purified by silica-gel column chromatography to afford the product **3xa**.

2-Phenyl-3-vinyloctadecan-2-ol (3ba): 1b (190 μL, 0.6 mmol, 3.0 equiv) and **2a** (24 μL, 0.2 mmol, 1.0 equiv) were employed as starting materials. Purification of the crude product by silica-gel column chromatography (eluent: hexane/ethyl acetate, 50:1) afforded **3ba** as colorless oil (58 mg, 155 μmol, 74%, dr = 1.2:1). IR (neat): 3467, 2924, 2853, 1637, 1465, 1375, 1065, 913, 759, 700 cm^{-1}; ^{1}H NMR (400 MHz, CDCl$_3$) δ: 7.44-7.21 (m, 5H), 5.64-5.52 (m, 1H), 5.21-5.05 (m, 2H), 2.29-2.23 (m, 1H), 1.54/1.51 (s, 3H), 1.34-1.02 (m, 28H), 0.88 (t, J = 7.0 Hz, 3H) ppm; ^{13}C NMR (100 MHz, CDCl$_3$) δ: 147.2, 146.5, 138.9, 138.6, 127.84, 127.76, 126.6, 126.3, 126.0, 125.2, 118.8, 118.1, 76.0, 75.5, 56.6, 55.6, 31.9, 29.7, 29.64, 29.60, 29.57, 29.52, 29.49, 29.48, 29.41, 29.38, 29.35, 28.9, 28.6, 28.5, 27.8, 27.7, 25.4, 22.7, 14.1 ppm; HRMS (EI) *m/z* calcd. for $C_{26}H_{42}$ [M-H$_2$O]$^+$: 354.3287, found: 354.3282.

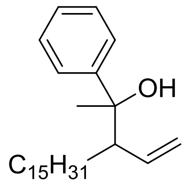

3ba

2,6-Diphenyl-3-vinylhexan-2-ol (3ca): 1c (96 mg, 0.6 mmol, 3.0 equiv) and **2a** (23 μL, 0.2 mmol, 1.0 equiv) were employed as starting materials. Purification of the crude product by silica-gel column chromatography (eluent: hexane/ethyl acetate, 40:1 to 10:1) afforded **3ca** as colorless oil (36 mg, 140 μmol, 67%, dr = 1.4:1). IR (neat): 3466, 3025, 2930, 2857, 1602, 1494, 1446, 914, 749, 700 cm^{-1}; ^{1}H NMR (400 MHz, CDCl$_3$) δ: 7.44-7.04 (m, 1H), 5.66-5.51 (m, 1H), 5.21-5.05 (m, 2H), 2.56-2.49 (m,

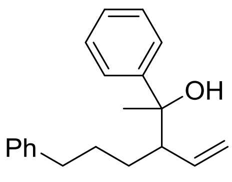

3ca

1H), 2.43-2.36 (m, 1H), 2.35-2.29 (m, 1H), 1.54/1.51 (s, 3H), 1.47-1.24 (m, 4H) ppm; ^{13}C NMR (100 MHz, $CDCl_3$) δ: 147.0, 146.4, 142.6, 142.5, 138.6, 138.3, 128.3, 128.2, 127.9, 127.8, 126.7, 126.4, 125.9, 125.5, 125.2, 119.1, 118.3, 76.0, 75.5, 56.5, 55.5, 35.7, 35.6, 29.8, 29.7, 28.9, 28.4, 28.2, 25.5 ppm; HRMS (ESI) *m/z* calcd. for $C_{20}H_{24}ONa$ $[M+Na]^+$: 303.1719, found: 303.1727.

(D) Copies of ^{1}H NMR and ^{13}C NMR spectra

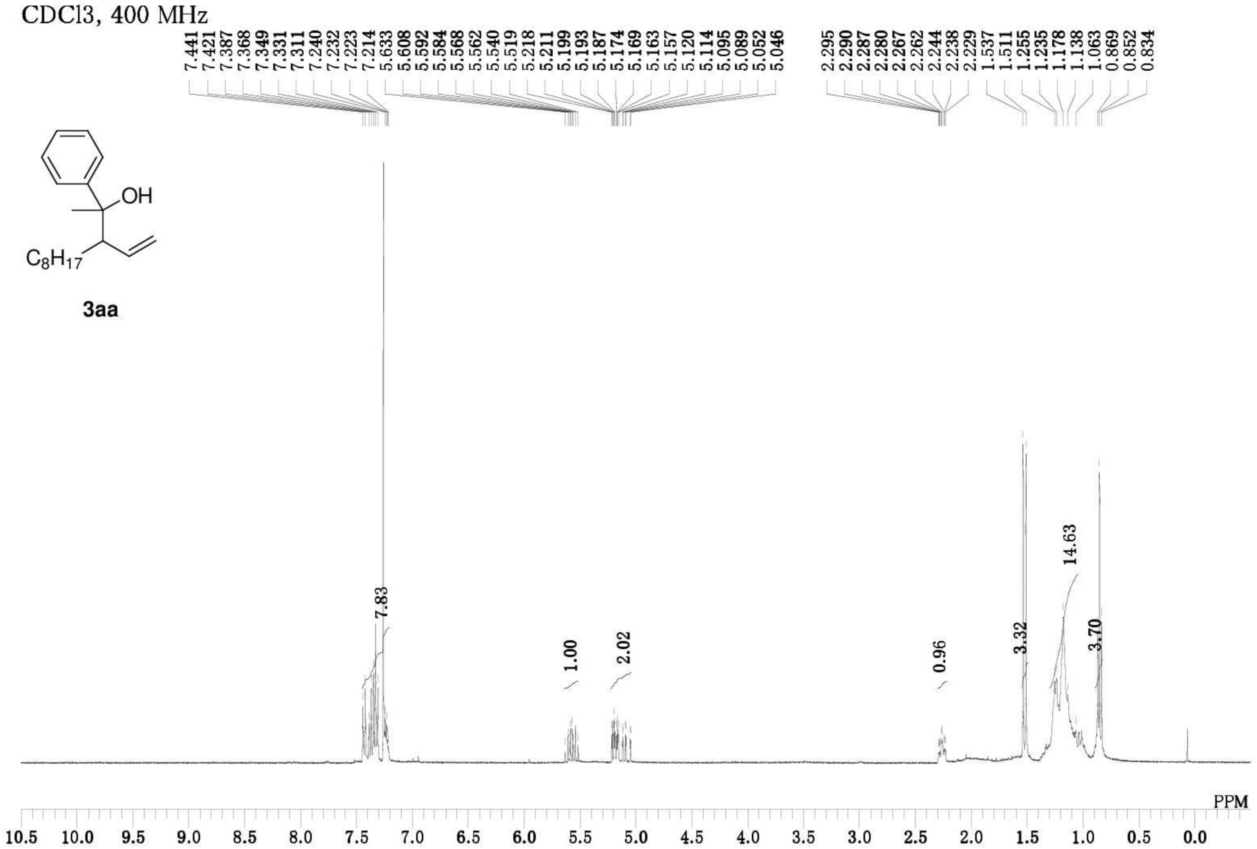

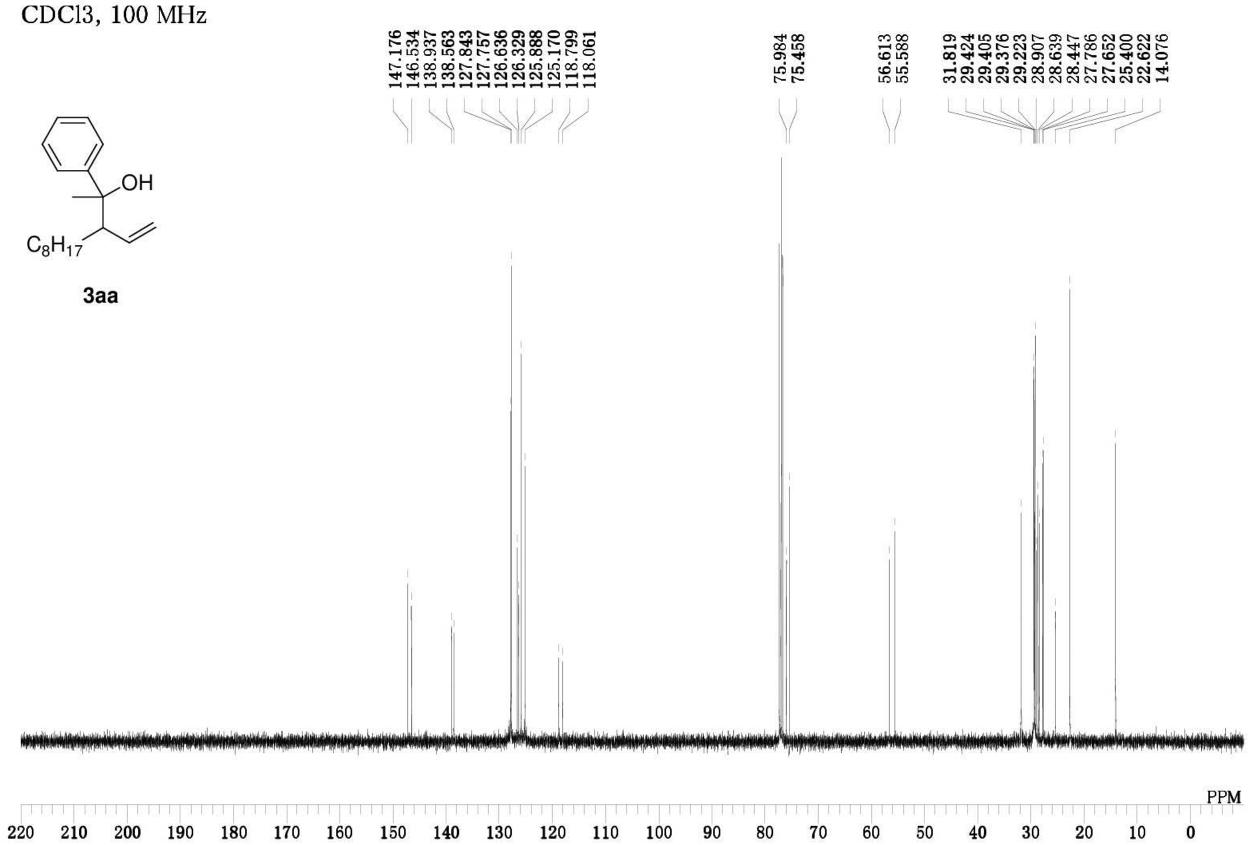

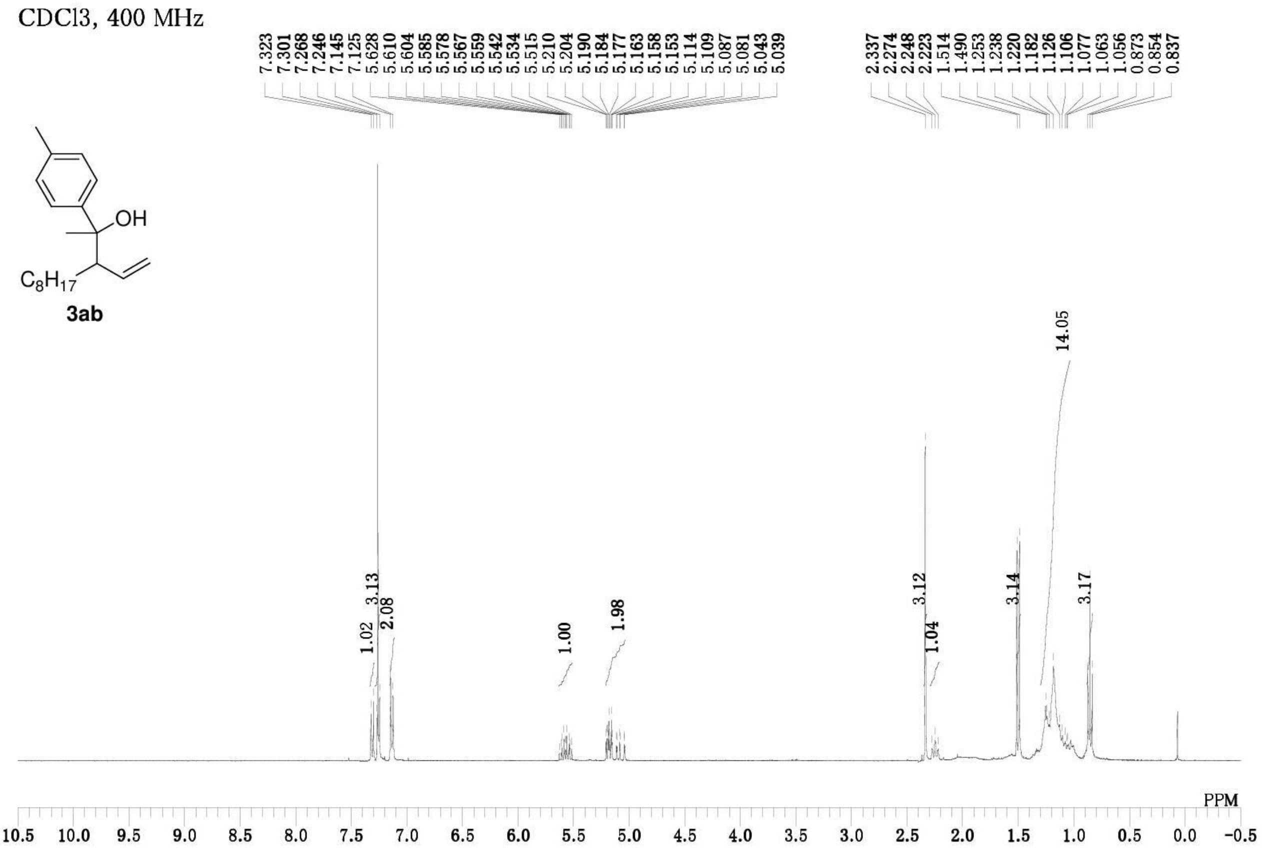
CDCl3, 400 MHz
7.323 7.301 7.268 7.246 7.145 7.125 5.628 5.610 5.604 5.585 5.578 5.567 5.559 5.542 5.534 5.515 5.210 5.204 5.190 5.184 5.177 5.163 5.158 5.153 5.114 5.109 5.087 5.081 5.043 5.039
2.337 2.274 2.248 2.223 1.514 1.490 1.253 1.238 1.220 1.182 1.126 1.106 1.077 1.063 1.056 0.873 0.854 0.837
OH
C8H17
3ab
14.05
1.02 3.13 2.08 1.00 1.98 3.12 1.04 3.14 3.17
PPM
10.5 10.0 9.5 9.0 8.5 8.0 7.5 7.0 6.5 6.0 5.5 5.0 4.5 4.0 3.5 3.0 2.5 2.0 1.5 1.0 0.5 0.0 −0.5

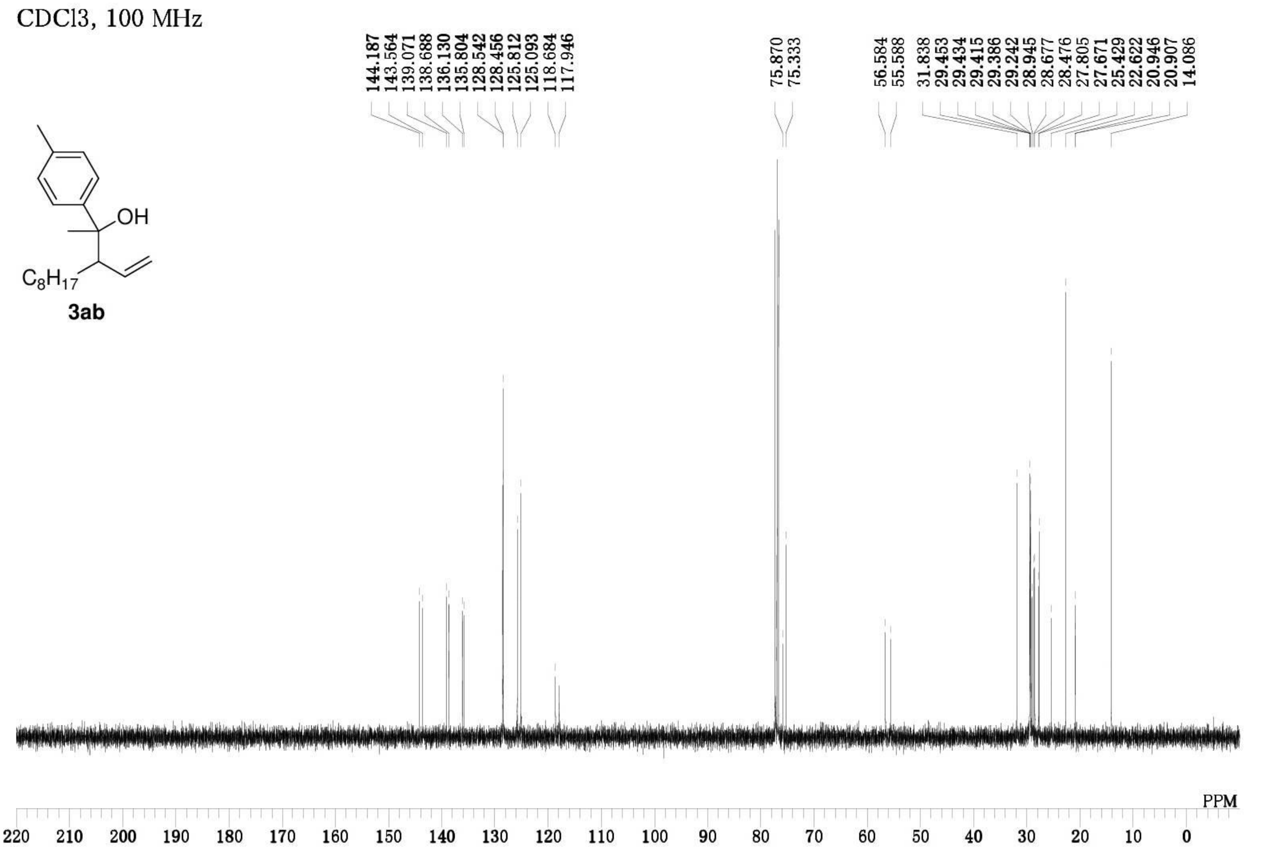
CDCl3, 100 MHz
144.187 143.564 139.071 138.688 136.130 135.804 128.542 128.456 125.812 125.093 118.684 117.946
75.870 75.333
56.584 55.588 31.838 29.453 29.434 29.415 29.386 29.242 28.945 28.677 28.476 27.805 27.671 25.429 22.622 20.946 20.907 14.086
OH
C8H17
3ab
PPM
220 210 200 190 180 170 160 150 140 130 120 110 100 90 80 70 60 50 40 30 20 10 0

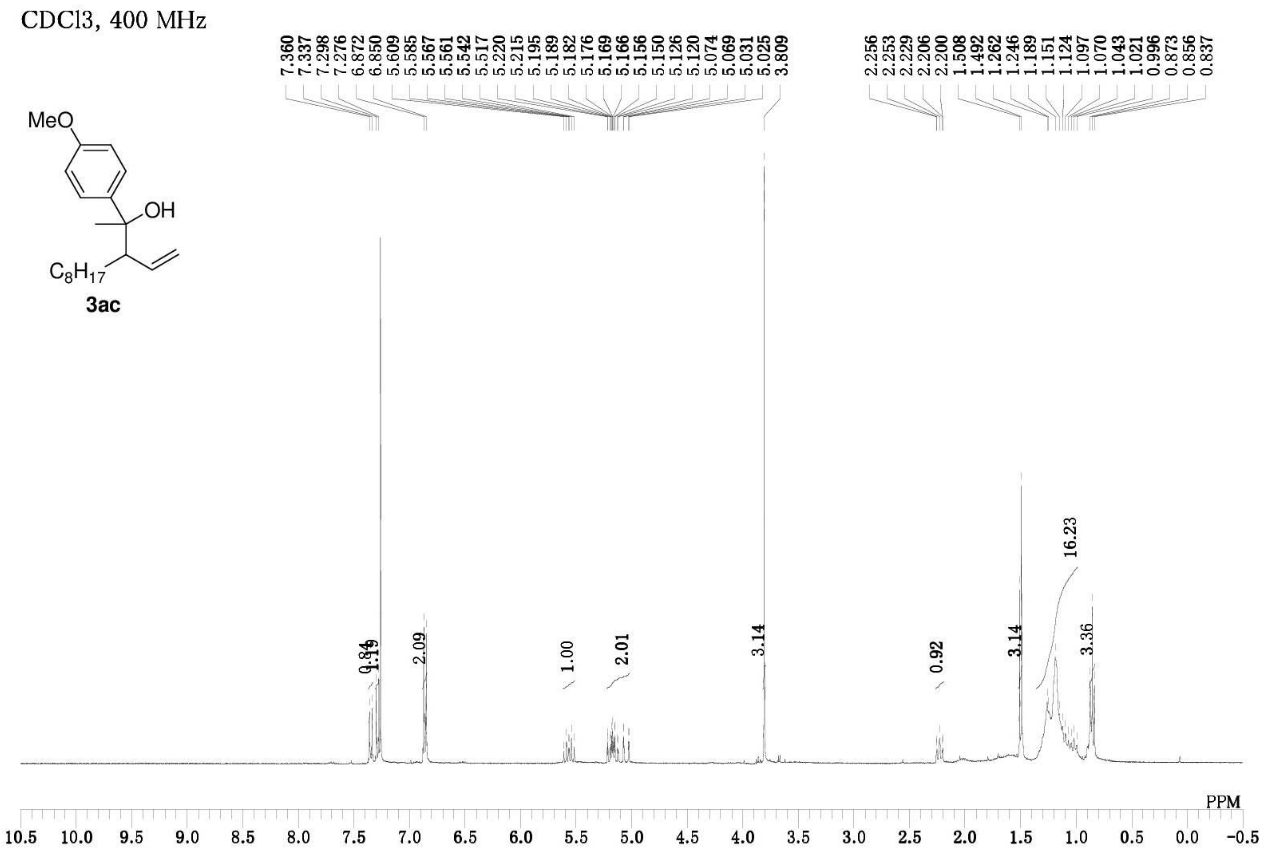
CDCl3, 400 MHz
3ac

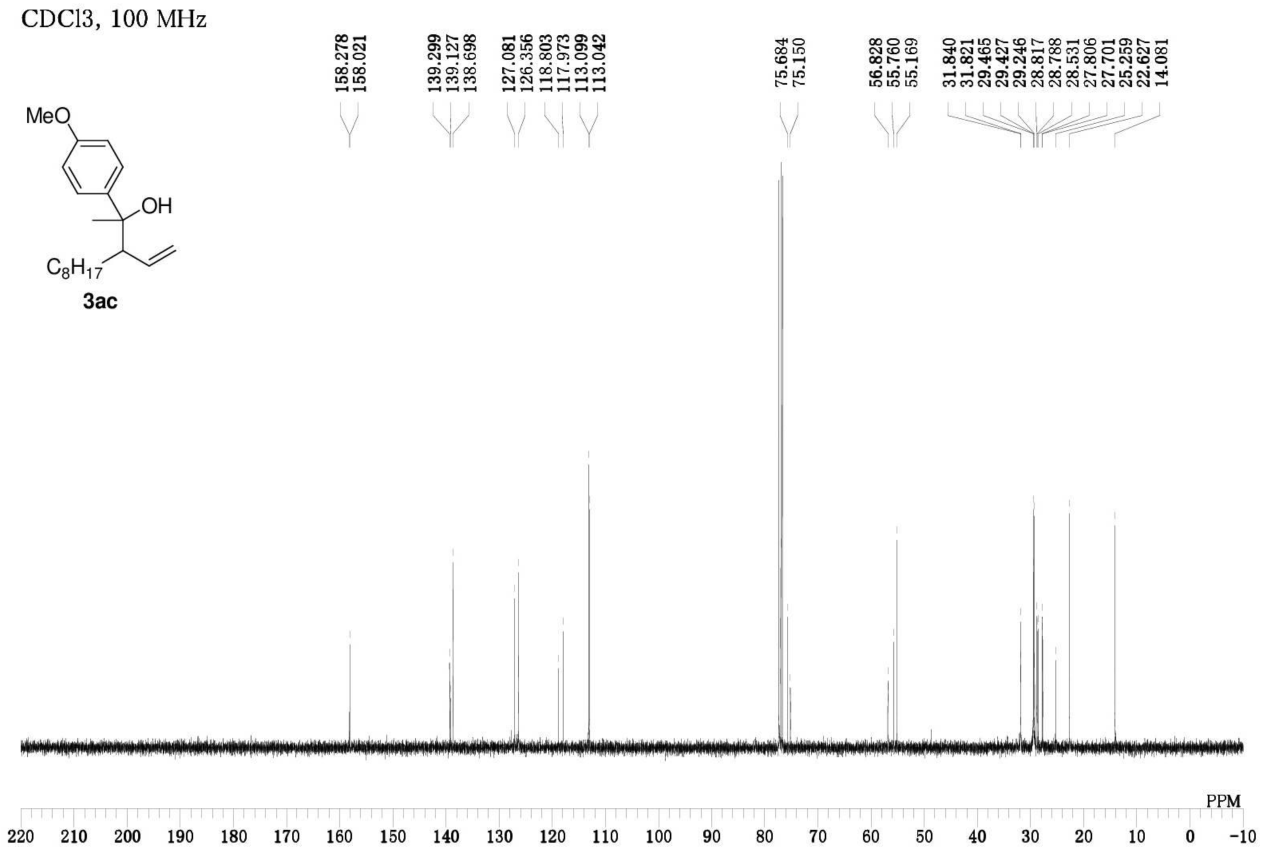
CDCl3, 100 MHz
3ac

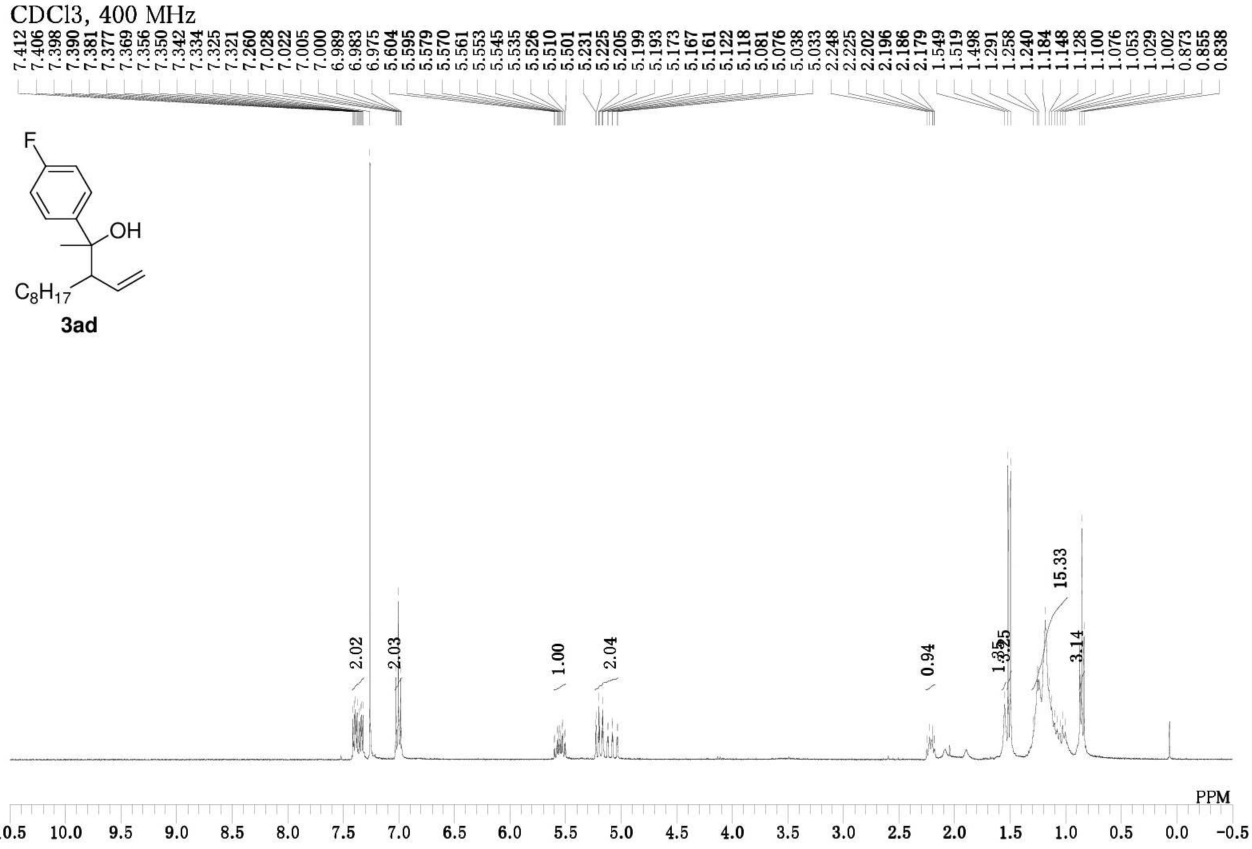
CDCl3, 400 MHz
7.412 7.406 7.398 7.390 7.381 7.377 7.369 7.356 7.350 7.342 7.334 7.325 7.321 7.260 7.028 7.022 7.005 7.000 6.989 6.983 6.975 5.604 5.595 5.579 5.570 5.561 5.553 5.545 5.535 5.526 5.510 5.501 5.231 5.225 5.205 5.199 5.193 5.173 5.167 5.161 5.122 5.118 5.081 5.076 5.038 5.033 2.248 2.225 2.202 2.196 2.186 2.179 1.549 1.519 1.498 1.291 1.258 1.240 1.184 1.148 1.128 1.100 1.076 1.053 1.029 1.002 0.873 0.855 0.838
F
OH
C_8H_{17}
3ad
2.02
2.03
1.00
2.04
0.94
15.33
3.14
PPM
10.5 10.0 9.5 9.0 8.5 8.0 7.5 7.0 6.5 6.0 5.5 5.0 4.5 4.0 3.5 3.0 2.5 2.0 1.5 1.0 0.5 0.0 −0.5

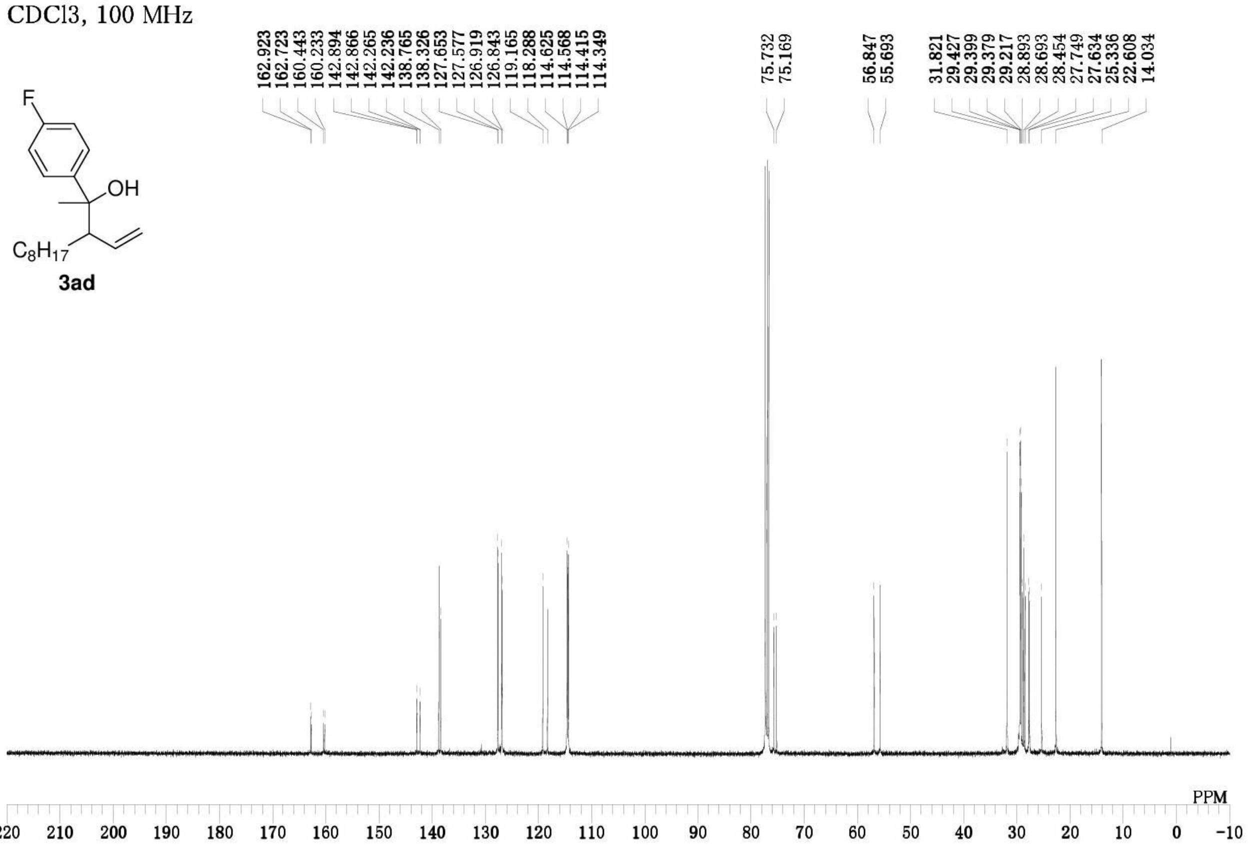
CDCl3, 100 MHz
162.923 162.723 160.443 160.233 142.894 142.866 142.265 142.236 138.765 138.326 127.653 127.577 126.919 126.843 119.165 118.288 114.625 114.568 114.415 114.349
75.732 75.169
56.847 55.693
31.821 29.427 29.399 29.379 29.217 28.893 28.693 28.454 27.749 27.634 25.336 22.608 14.034
F
OH
C_8H_{17}
3ad
PPM
220 210 200 190 180 170 160 150 140 130 120 110 100 90 80 70 60 50 40 30 20 10 0 −10

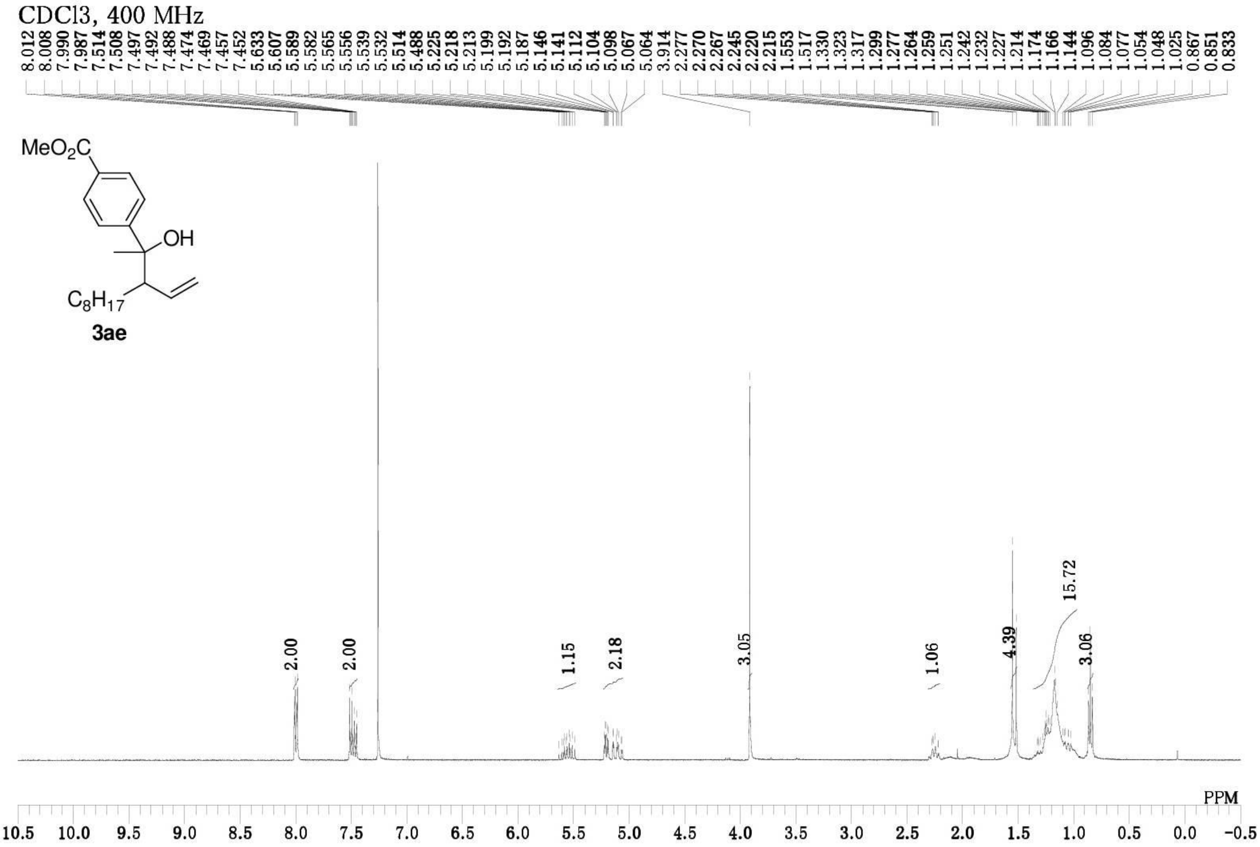
CDCl3, 400 MHz
8.012 8.008 7.990 7.987 7.514 7.508 7.497 7.492 7.488 7.474 7.469 7.457 7.452 5.633 5.607 5.589 5.582 5.565 5.556 5.539 5.532 5.514 5.488 5.225 5.218 5.213 5.199 5.192 5.187 5.146 5.141 5.112 5.104 5.098 5.067 5.064 3.914 2.277 2.270 2.267 2.245 2.220 2.215 1.553 1.517 1.330 1.323 1.317 1.299 1.277 1.264 1.259 1.251 1.242 1.232 1.227 1.214 1.174 1.166 1.144 1.096 1.084 1.077 1.054 1.048 1.025 0.867 0.851 0.833
MeO2C
OH
C8H17
3ae
2.00
2.00
1.15
2.18
3.05
1.06
4.39
15.72
3.06
PPM
10.5 10.0 9.5 9.0 8.5 8.0 7.5 7.0 6.5 6.0 5.5 5.0 4.5 4.0 3.5 3.0 2.5 2.0 1.5 1.0 0.5 0.0 −0.5

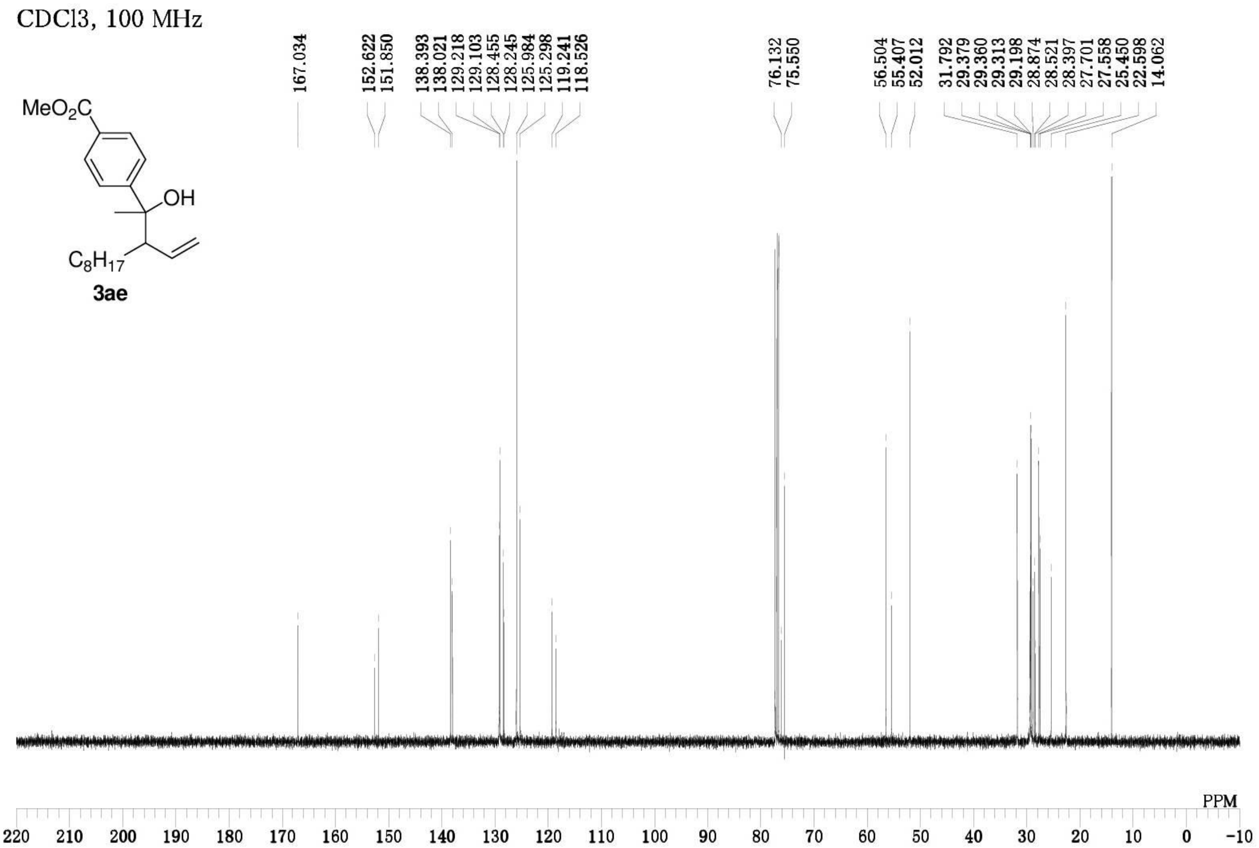
CDCl3, 100 MHz
167.034
152.622 151.850
138.393 138.021 129.218 129.103 128.455 128.245 125.984 125.298 119.241 118.526
76.132 75.550
56.504 55.407 52.012
31.792 29.379 29.360 29.313 29.198 28.874 28.521 28.397 27.701 27.558 25.450 22.598 14.062
MeO2C
OH
C8H17
3ae
PPM
220 210 200 190 180 170 160 150 140 130 120 110 100 90 80 70 60 50 40 30 20 10 0 −10

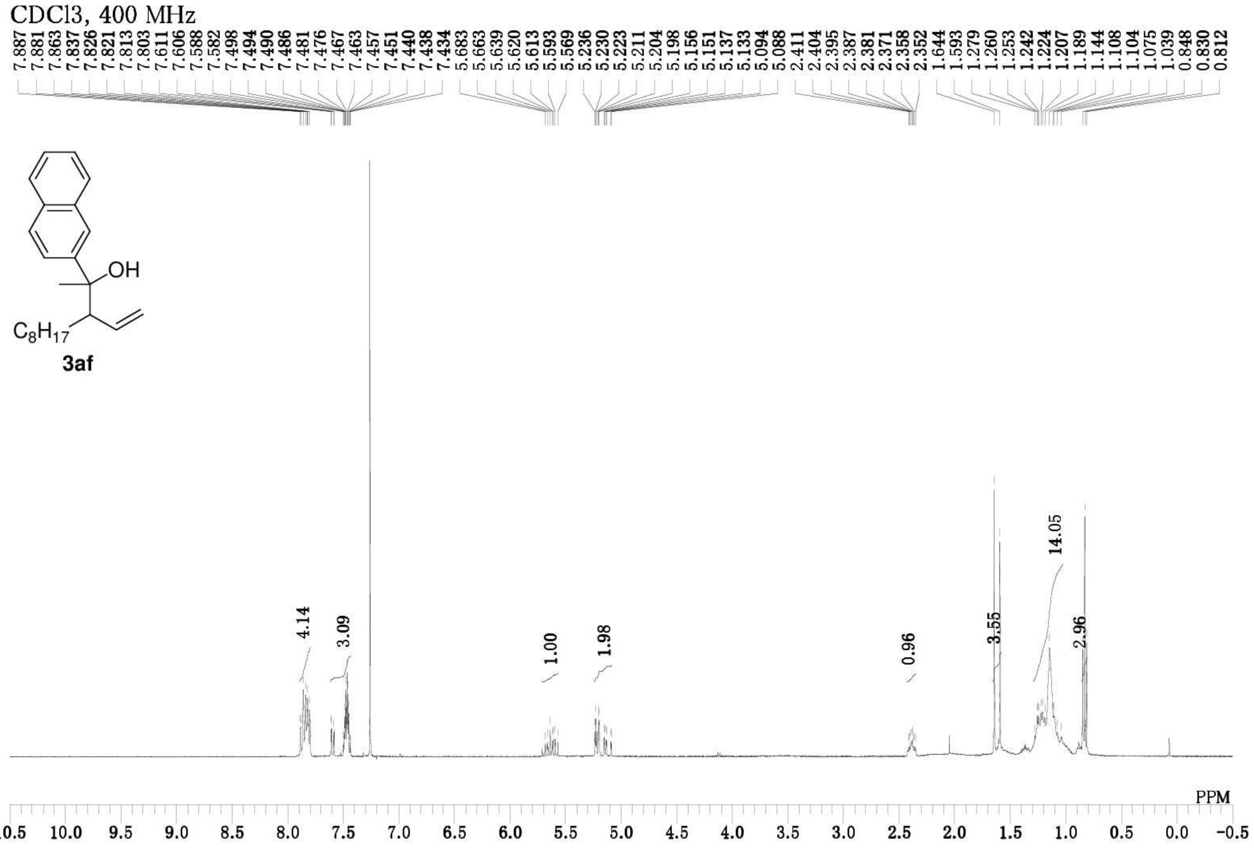
CDCl3, 400 MHz
7.887 7.881 7.863 7.837 7.826 7.821 7.813 7.803 7.611 7.606 7.588 7.582 7.498 7.494 7.490 7.486 7.481 7.476 7.467 7.463 7.457 7.451 7.440 7.438 7.434 5.683 5.663 5.639 5.620 5.613 5.593 5.569 5.236 5.230 5.223 5.211 5.204 5.198 5.156 5.151 5.137 5.133 5.094 5.088 2.411 2.404 2.395 2.387 2.381 2.371 2.358 2.352 1.644 1.593 1.279 1.260 1.253 1.242 1.224 1.207 1.189 1.144 1.108 1.104 1.075 1.039 0.848 0.830 0.812
OH
C8H17
3af
4.14
3.09
1.00
1.98
0.96
3.55
14.05
2.96
PPM
10.5 10.0 9.5 9.0 8.5 8.0 7.5 7.0 6.5 6.0 5.5 5.0 4.5 4.0 3.5 3.0 2.5 2.0 1.5 1.0 0.5 0.0 −0.5

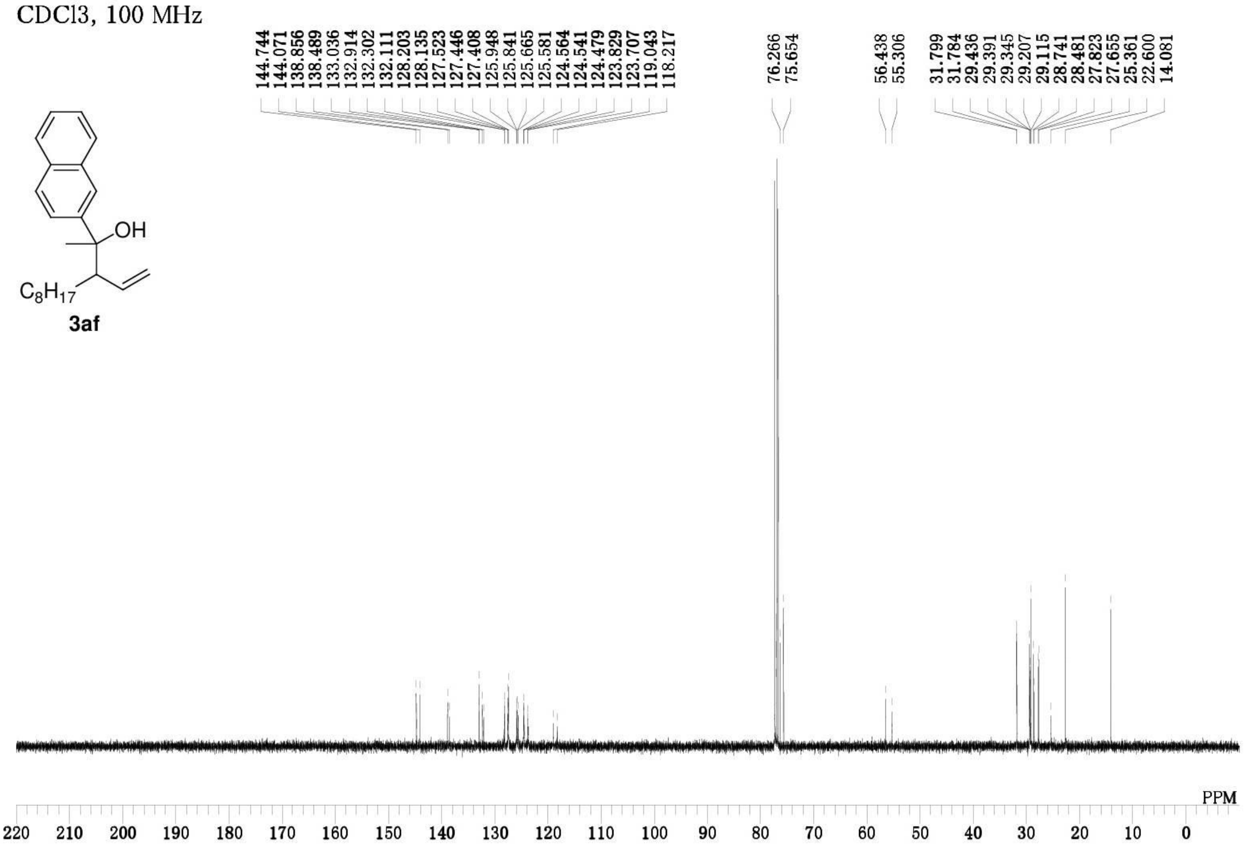
CDCl3, 100 MHz
144.744 144.071 138.856 138.489 133.036 132.914 132.302 132.111 128.203 128.135 127.523 127.446 127.408 125.948 125.841 125.665 125.581 124.564 124.541 124.479 123.829 123.707 119.043 118.217
76.266 75.654
56.438 55.306
31.799 31.784 29.436 29.391 29.345 29.207 29.115 28.741 28.481 27.823 27.655 25.361 22.600 14.081
OH
C8H17
3af
PPM
220 210 200 190 180 170 160 150 140 130 120 110 100 90 80 70 60 50 40 30 20 10 0

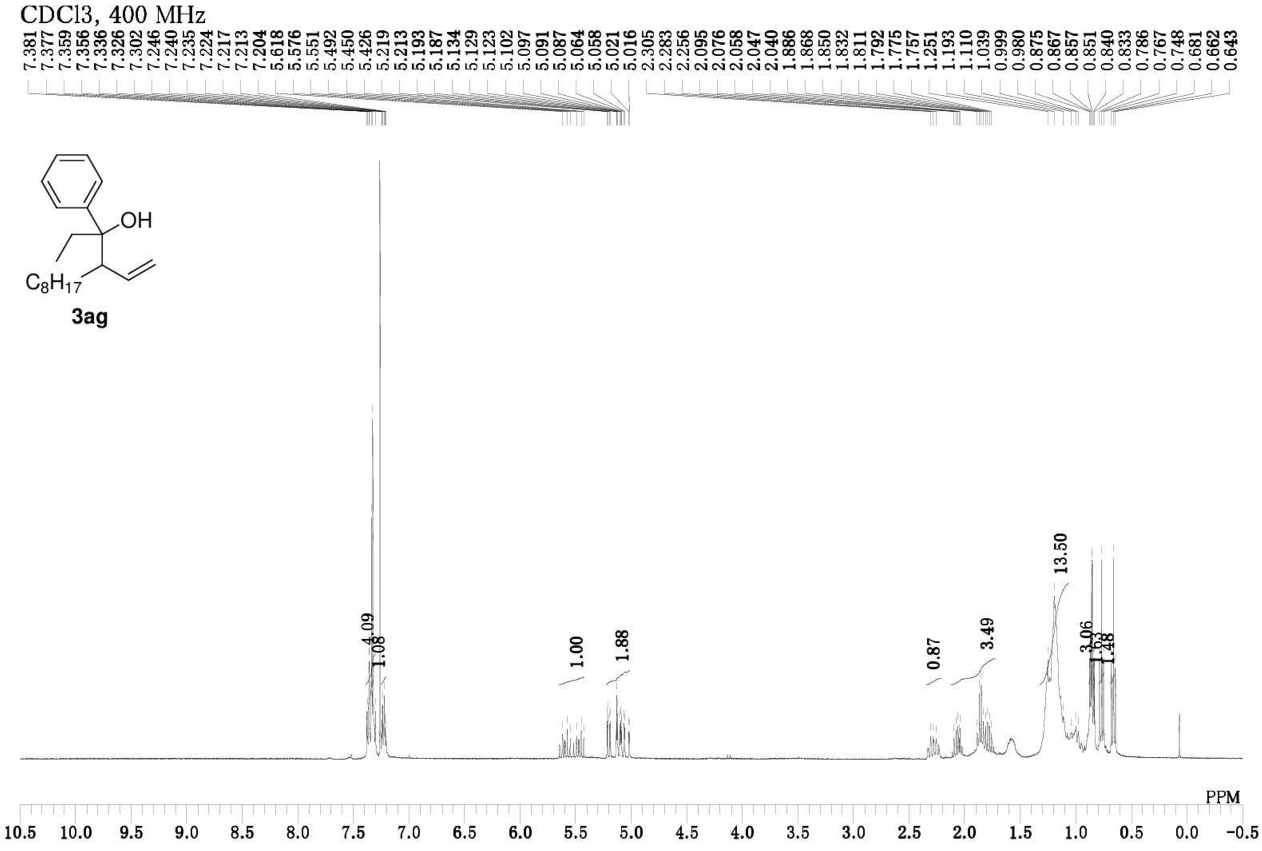
CDCl3, 400 MHz
7.381
7.377
7.359
7.356
7.336
7.326
7.302
7.246
7.240
7.235
7.224
7.217
7.213
7.204
5.618
5.576
5.551
5.492
5.450
5.426
5.219
5.213
5.193
5.187
5.134
5.129
5.123
5.102
5.097
5.091
5.087
5.064
5.058
5.021
5.016
2.305
2.283
2.256
2.095
2.076
2.058
2.047
2.040
1.886
1.868
1.850
1.832
1.811
1.792
1.775
1.757
1.251
1.193
1.110
1.039
0.999
0.980
0.875
0.867
0.857
0.851
0.840
0.833
0.786
0.767
0.748
0.681
0.662
0.643
OH
C8H17
3ag
4.09
1.08
1.00
1.88
0.87
3.49
13.50
3.06
1.48
PPM

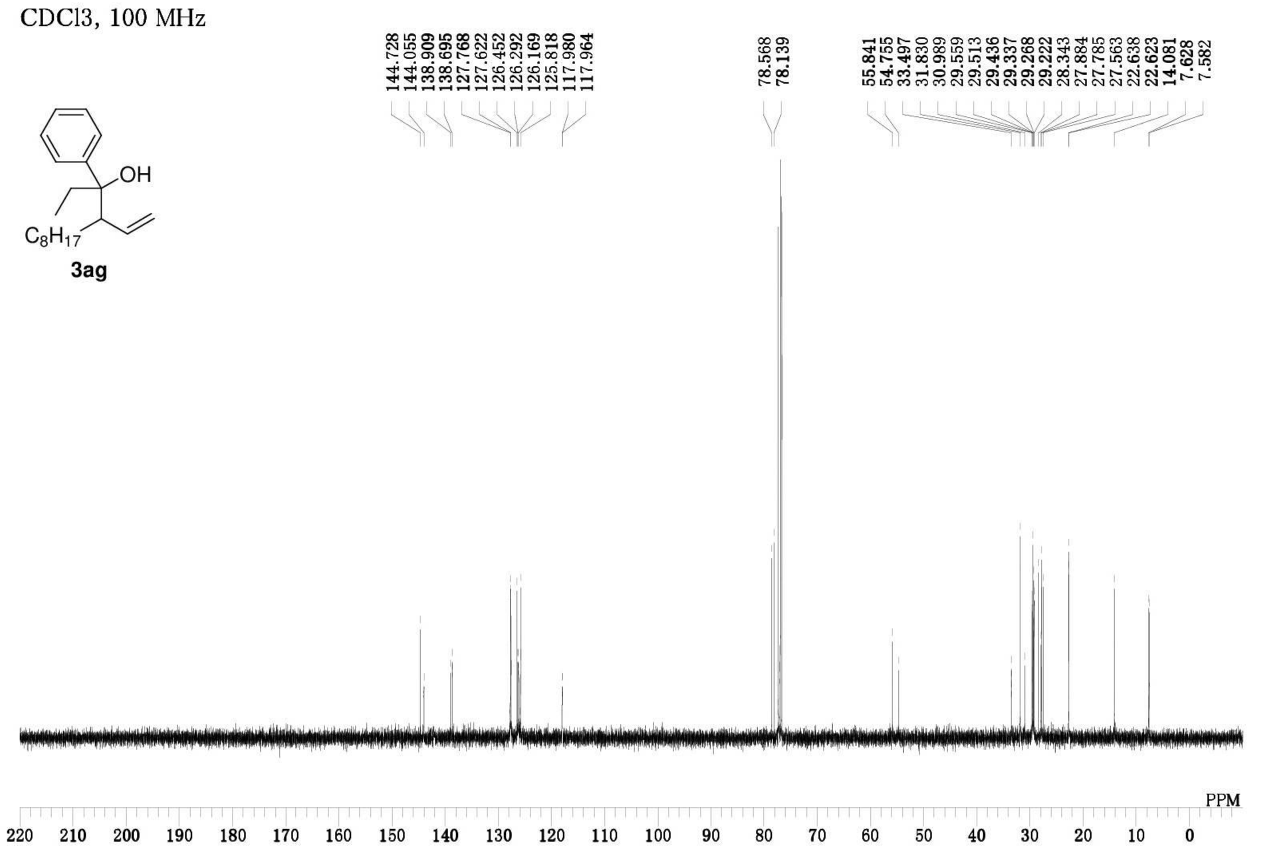
CDCl3, 100 MHz
144.728
144.055
138.909
138.695
127.768
127.622
126.452
126.292
126.169
125.818
117.980
117.964
78.568
78.139
55.841
54.755
33.497
31.830
30.989
29.559
29.513
29.436
29.337
29.268
29.222
28.343
27.884
27.785
27.563
22.638
22.623
14.081
7.628
7.582
OH
C8H17
3ag
PPM

CDCl3, 400 MHz

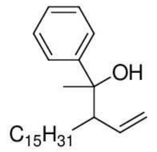

3ba

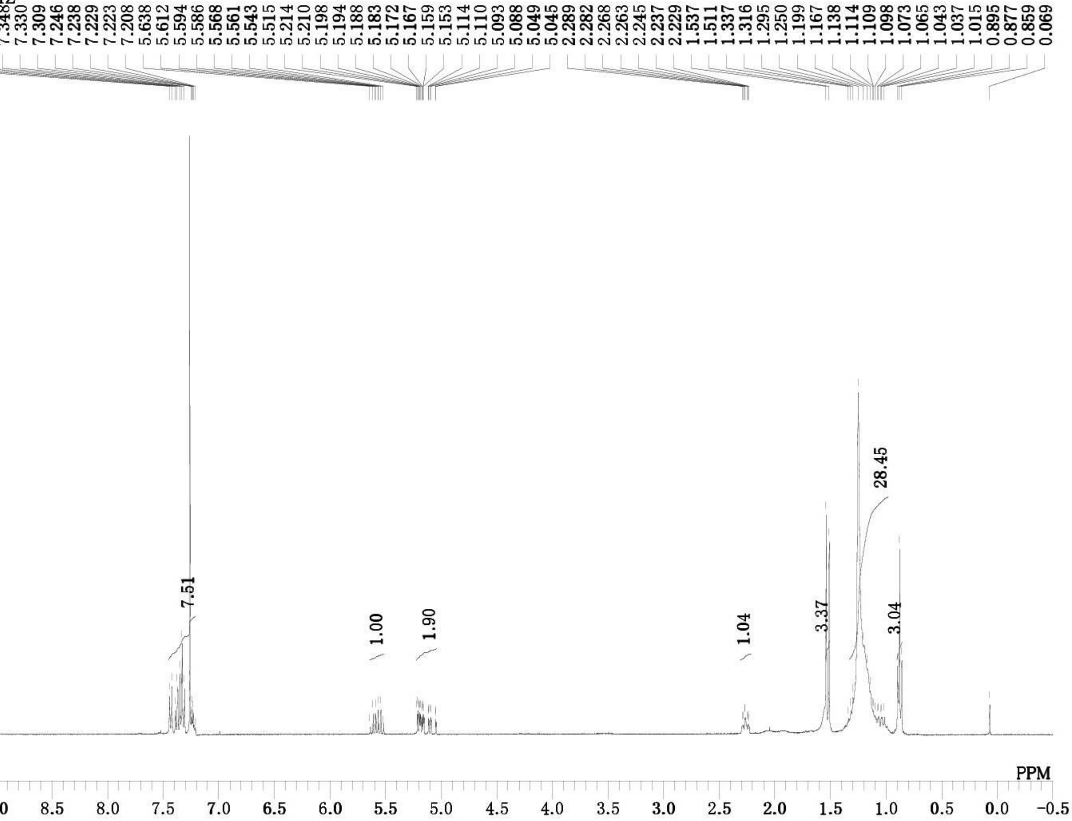

CDCl3, 100 MHz

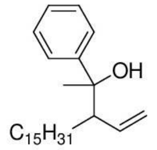

3ba

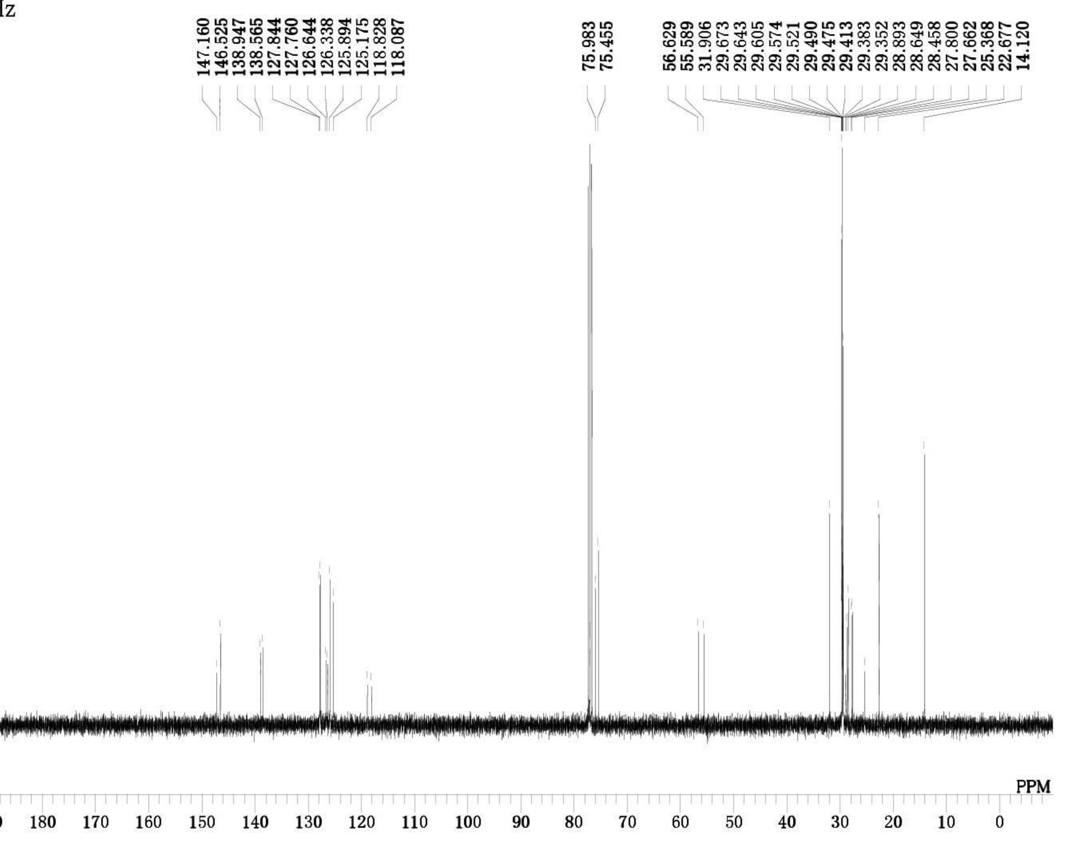

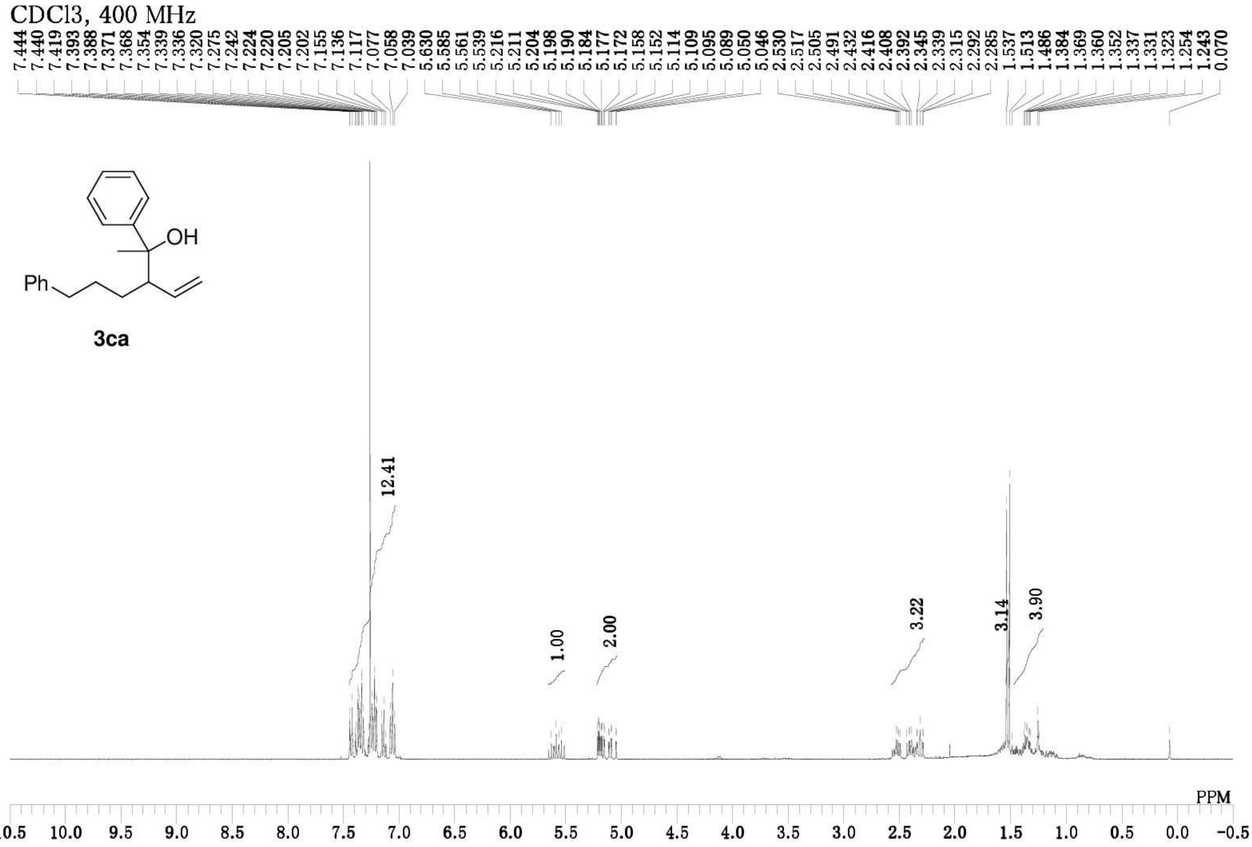
CDCl3, 400 MHz
7.444 7.440 7.419 7.393 7.388 7.371 7.368 7.354 7.339 7.336 7.320 7.275 7.242 7.224 7.220 7.205 7.202 7.155 7.136 7.117 7.077 7.058 7.039 5.630 5.585 5.561 5.539 5.216 5.211 5.204 5.198 5.190 5.184 5.177 5.172 5.158 5.152 5.114 5.109 5.095 5.089 5.050 5.046 2.530 2.517 2.505 2.491 2.432 2.416 2.408 2.392 2.345 2.339 2.315 2.292 2.285 1.537 1.513 1.486 1.384 1.369 1.360 1.352 1.337 1.331 1.323 1.254 1.243 0.070
OH
Ph
3ca
12.41
1.00
2.00
3.22
3.14
3.90
PPM
10.5 10.0 9.5 9.0 8.5 8.0 7.5 7.0 6.5 6.0 5.5 5.0 4.5 4.0 3.5 3.0 2.5 2.0 1.5 1.0 0.5 0.0 −0.5

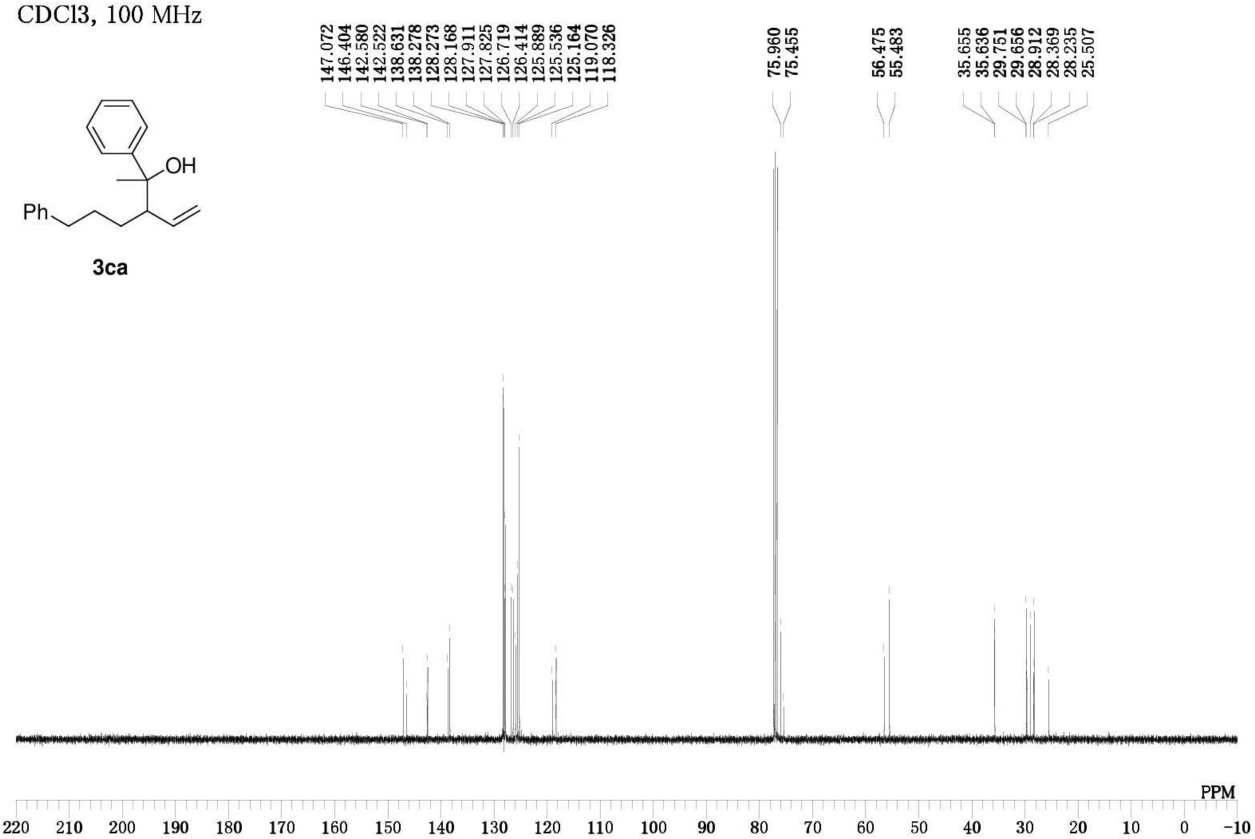
CDCl3, 100 MHz
147.072 146.404 142.580 142.522 138.631 138.278 128.273 128.168 127.911 127.825 126.719 126.414 125.889 125.536 125.164 119.070 118.326
75.960 75.455
56.475 55.483
35.655 35.636 29.751 29.656 28.912 28.369 28.235 25.507
OH
Ph
3ca
PPM
220 210 200 190 180 170 160 150 140 130 120 110 100 90 80 70 60 50 40 30 20 10 0 −10
